# Differential roles of STAT1 and STAT2 in the sensitivity of JAK2V617F- vs. BCR-ABL-positive cells to interferon alpha

**DOI:** 10.1186/s13045-019-0722-9

**Published:** 2019-04-02

**Authors:** Claudia Schubert, Manuel Allhoff, Stefan Tillmann, Tiago Maié, Ivan G. Costa, Daniel B. Lipka, Mirle Schemionek, Kristina Feldberg, Julian Baumeister, Tim H. Brümmendorf, Nicolas Chatain, Steffen Koschmieder

**Affiliations:** 1 Department of Hematology, Oncology, Hemostaseology, and Stem Cell Transplantation, Faculty of Medicine, RWTH Aachen University, Pauwelsstr 30, 52074 Aachen, Germany; 2 Institute for Computational Genomics, Faculty of Medicine, RWTH Aachen University, Aachen, Germany; 3 Regulation of Cellular Differentiation Group, Division of Epigenomics and Cancer Risk Factors, German Cancer Research Center (DKFZ), Heidelberg, Germany

**Keywords:** IFNa, BCR-ABL, JAK2V617F, MPN, Polycythemia vera, Chronic myeloid leukemia, STAT factors, ISG, CRISPR/Cas9

## Abstract

**Background:**

Interferon alpha (IFNa) monotherapy is recommended as the standard therapy in polycythemia vera (PV) but not in chronic myeloid leukemia (CML). Here, we investigated the mechanisms of IFNa efficacy in JAK2V617F- vs. BCR-ABL-positive cells.

**Methods:**

Gene expression microarrays and RT-qPCR of PV vs. CML patient PBMCs and CD34+ cells and of the murine cell line 32D expressing JAK2V617F or BCR-ABL were used to analyze and compare interferon-stimulated gene (ISG) expression. Furthermore, using CRISPR/Cas9n technology, targeted disruption of STAT1 or STAT2, respectively, was performed in 32D-BCR-ABL and 32D-JAK2V617F cells to evaluate the role of these transcription factors for IFNa efficacy. The knockout cell lines were reconstituted with STAT1, STAT2, STAT1Y701F, or STAT2Y689F to analyze the importance of wild-type and phosphomutant STATs for the IFNa response. ChIP-seq and ChIP were performed to correlate histone marks with ISG expression.

**Results:**

Microarray analysis and RT-qPCR revealed significant upregulation of ISGs in 32D-JAK2V617F but downregulation in 32D-BCR-ABL cells, and these effects were reversed by tyrosine kinase inhibitor (TKI) treatment. Similar expression patterns were confirmed in human cell lines, primary PV and CML patient PBMCs and CD34+ cells, demonstrating that these effects are operational in patients. IFNa treatment increased *Stat1*, *Stat2*, and *Irf9* mRNA as well as pY-STAT1 in all cell lines; however, viability was specifically decreased in 32D-JAK2V617F. STAT1 or STAT2 knockout and reconstitution with wild-type or phospho-deficient STAT mutants demonstrated the necessity of STAT2 for IFNa-induced STAT1 phosphorylation in BCR-ABL- but not in JAK2V617F-expressing cells. STAT1 was essential for IFNa activity in both BCR-ABL- and JAK2V617F-positive cells. Furthermore, ChIP experiments demonstrate higher repressive and lower active chromatin marks at the promoters of ISGs in BCR-ABL-expressing cells.

**Conclusions:**

JAK2V617F but not BCR-ABL sensitizes MPN cells to interferon, and this effect was dependent on STAT1. Moreover, STAT2 is a survival factor in BCR-ABL- and JAK2V617F-positive cells but an IFNa-sensitizing factor solely in 32D-JAK2V617F cells by upregulation of STAT1 expression.

**Electronic supplementary material:**

The online version of this article (10.1186/s13045-019-0722-9) contains supplementary material, which is available to authorized users.

## Background

Myeloproliferative neoplasms (MPN) are chronic malignancies that are closely associated with specific oncogenes such as BCR-ABL, present in chronic myeloid leukemia (CML) [1], and JAK2V617F, present in polycythemia vera (PV) [2], essential thrombocythemia (ET), and primary myelofibrosis (PMF) [3].

Since 2001, imatinib is the approved standard therapy for treatment of CML that targets differentiated cells [4, 5]. Imatinib treatment induces a complete cytogenetic response and increases survival and freedom from progression [6]. The JAK1 and JAK2 inhibitor ruxolitinib was found to reduce spleen volume and to improve disease-related symptoms in patients with myelofibrosis [7, 8] and hydroxyurea-intolerant or hydroxyurea-resistant PV [9]. However, both imatinib and ruxolitinib do not eradicate CML or MPN stem cells in the majority of cases [10, 11]. Conversely, both CML and PV stem cells have recently been demonstrated to be targeted by interferon alpha (IFNa) [12–14], but it is not well understood which pathways are responsible for the IFNa response in both diseases.

IFNa induces phosphorylation of signal transducers and activators of transcription 1 (STAT1) and 2 (STAT2) that assemble with interferon-regulatory-factor 9 (IRF9) to form the IFN-stimulated gene factor 3 (ISGF3) complex, which binds to ISRE (IFN-stimulated response element) consensus sequences of their target genes [15]. Although the STAT1-dependent pathway may be the dominant signaling cascade to mediate the antiproliferative effect of IFNa, STAT3 and STAT5 play a role in IFN signaling as well, if only in case of suppressed or abrogated STAT1 signaling [16]. This mechanism may be relevant in CML, since it was shown that cells of IFNa-resistant patients lack *STAT1* expression [17] and in PV, when STAT1 function is inhibited by ruxolitinib. Moreover, the JAK/STAT-negative regulator suppressor of cytokine signaling 3 (SOCS3) was upregulated in 50% of CML patients that did not respond to IFNa [18], and BCR-ABL was shown to suppress the expression of IFNa target genes, thereby modulating the IFNa-mediated response [19, 20].

In addition to the JAK/STAT pathway, other signaling pathways are activated by IFNa, such as the p38 mitogen-activated protein kinase (MAPK) pathway [21]. Activation of p38 MAPK has been shown to be responsible for IFNa-induced growth inhibition in IFNa-sensitive CML cells [22] as well as in primary CD34+ cells from PV patients [23]. Furthermore, ULK1 as a trigger of IFNa-induced p38 MAPK signaling in Ph^+^ MPN cells and MEK/ERK-mediated Mnk activation in JAK2V617F-positive cells were described to be required for IFNa-induced growth inhibition [22–25]. Importantly, BCR-ABL expression leads to the degradation of the IFNa receptor (IFNAR) by ubiquitination that could be counteracted by pretreatment of CML cells with imatinib, restoring IFNa sensitivity [26]. But even though IFNAR is downregulated during IFNa therapy [27], this was found to be independent from IFNa responsiveness. Thus, while some IFNa target genes and proteins may be shared between CML and PV, it remains unclear whether the same mechanisms are important for IFNa-induced biologic effects.

In this study, we analyzed the cellular and molecular response of BCR-ABL- and JAK2V617F-expressing cells to IFNa, aiming to clarify the roles of STAT1 and STAT2 in IFNa monotherapy in CML and PV.

## Methods

### Patient samples

Primary patient samples were obtained from patients of the Department of Hematology, Oncology, Hemostaseology, and Stem Cell Transplantation at RWTH Aachen University after written informed consent, as approved by the local ethics committee (EK 127/12 and EK 206/09). The CML patients were newly diagnosed. None of the PV patients had received ruxolitinib or IFNa before sample preparation.

### DNA constructs and vectors

The cDNA of *Stat1* was amplified from cDNA of 32D-JAK2V617F, and *Stat2* cDNA was ordered from GeneArt gene synthesis service of Thermo Fisher Scientific. Amplification was done with the primers listed in Additional file 1: Table S1. The PCR products were ligated into the pMSCV-IRES-RFP vector digested with the depicted restriction enzymes (NEB) (Additional file 1: Table S1).

Mutagenesis of *STAT1* and *STAT2* to substitute Y701 (*STAT1*) or Y689 (*STAT2*) to phenylalanine (F) was performed with the Quick change II XL site-directed mutagenesis kit (Agilent Technologies) or Q5 mutagenesis kit (NEB) according to the manufacturer’s protocol with primers listed in Additional file 1: Table S2. Successful mutagenesis was controlled by Sanger sequencing (Eurofins Genomics S.A., MWG-Biotech, Ebersberg, Germany).

### Cell culture

The production of viral particles and retroviral transduction of 32D cells was carried out as previously described [28, 29]. The vectors pMY-IRES-GFP-*Bcr-Abl*, *Jak2V617F*, or the empty vector (EV) were used for the production of virus. 32D-BCR-ABL or 32D-JAK2V617F cells grow IL-3 independent and can be selected by fluorescence-activated cell sorting (FACS) for GFP expression. After transduction with the pMSCV-IRES-RFP vectors for the overexpression of the STAT proteins, the cells were FACS-sorted for double positivity of GFP and RFP with an Aria cell sorter (BD Bioscience, Heidelberg, Germany).

The cell lines 32D, K562 (BCR-ABL positive), and HEL (homozygously JAK2V617F positive) were purchased from the German collection of microorganisms and cell cultures (DSMZ, Braunschweig, Germany) and cultured in RPMI-1640 medium with 10% FCS and 1% PenStrep. WEHI supernatant (10%) served as IL-3 source for culturing 32D cells.

### Real-time quantitative reverse transcriptase-PCR (RT-qPCR)

RNA isolation, cDNA transcription, and procedure of RT-qPCR were performed as previously described [28]. In general, 32D cells were grown in WEHI-supplemented (IL-3-source) RPMI medium. Sixteen hours before RNA isolation, 32D cells were washed twice and resuspended in IL-3-free medium. Expression was calculated as a percentage of *Gapdh* (Glycerinaldhyd-3-phosphate-dehydrogenase). The primers for the analysis of target genes are listed in Additional file 1: Table S3.

For microarray analysis, RNA from 32D-EV, 32D-BCR-ABL, or 32D-JAK2V617F cells was analyzed, using an Affymetrix mouse genome 430 2.0 array (Fisher Scientific, Life Technologies, Carlsbad, CA, USA).

### SDS-Page and immunoblotting

Western blot analysis was performed as previously described [28], and proteins were detected via chemiluminescence (Fusion SL, PeqLab, Erlangen, Germany). The used antibodies are listed in Additional file 1: Table S4.

### MTT assay

Cells were plated in triplicate (5 × 10^4^ cells/well) in WEHI-free medium. IFNa (Miltenyi Biotech; mouse IFNa, source: HEK293 cells; batches changed during the study) was diluted in cell culture medium (0–10^4^ U/ml). In case of combination treatment with TKI, the cells were treated with 0.1 to 1 μM TKI, as indicated, in addition to the different IFN concentrations in comparison to a DMSO control. The measurement of cell viability was performed 72 h later using MTT reagent (Sigma-Aldrich), and measurement of absorption was conducted at a wavelength of 550 nm. The metabolic process of tetrazolium reduction to formazan in our assay reflects the amount of viable cells, and this is therefore stated accordingly. The viability was calculated in reference to the control cells.

### Apoptosis staining

Apoptosis staining of cells after TKI (0.5 μM imatinib and 0.1 μM ruxolitinib) and/or IFNa treatment (10^2^ U/ml) was done with propidium iodide (Sigma-Aldrich). 1 × 10^6^ cells were treated for 72 h or 48 h and stained with Zombie-Aqua or PI, respectively, in a 1:1000 dilution. The samples were analyzed with a FACS Gallios (Beckmann Coulter, Krefeld, Germany).

### CRISPR/Cas9n triggered STAT1 or STAT2 knockout

32D-BCR-ABL-S1ko or S2ko and 32D-JAK2V617F-S1ko or S2ko cells were generated with CRISPR/Cas9n double nicking approach as described by Ran et al. [30]. In brief, two pairs of guide RNA (gRNA) were designed for excision of exon 5-7 in the *Stat1* and exon 9-20 in the *Stat2* gene (Additional file 1: Table S5; Additional file 2: Figure S1). gRNA oligonucleotides were cloned into a variant of the pX462 vector (Addgene plasmid # 62987), which was a gift from Feng Zhang (Addgene plasmid # 62987) [30]. Five micrograms of each gRNA plasmid, targeting STAT1 or STAT2, was electroporated into 32D-BCR-ABL or 32D-JAK2V617F cells using the NEON transfection system (1.350 V, 20 ms, 2 pulse, Thermo Fisher Scientific). Electroporated cells were selected by puromycin treatment (3 μg/ml) for 24 h. Again 24 h later, single cell dilutions were performed to obtain single clones.

### Chromatin immunoprecipitation

Chromatin immunoprecipitation (ChIP) experiments were conducted as previously described [31]. Antibodies and primers are listed in Additional file 1: Tables S6 and S7, respectively. Results were generated in at least two independent experiments.

Two different sets of H3K9ac ChIP-seq data were analyzed. One was published before [32] and included 32D-EV, 32D-BCR-ABL, and 32D-JAK2V617F cells. The other one was performed by our group in collaboration with the German Cancer Research Center (DKFZ) in Heidelberg in the Division of Epigenomics and Cancer Risk Factors and additionally included the corresponding TKI treatment. The detailed procedure is described in the supplementary information.

### Bioinformatic analyses

Analysis of expression arrays was based on Bioconductor, and analysis of ChIP-seq data was based on differential peak caller THOR [33] to detect peaks gained/lost between previously described pairs of conditions. Gene expression data from patients with BCR-ABL mutations and respective controls were obtained from Gene Expression Omnibus (GSE5550) [34]. The detailed analysis procedure is given in supplementary information. Gene set enrichment analysis (GSEA) was performed with the bionconductor package piano [35]. All gene sets from the Molecular Signatures Database (https://software.broadinstitute.org/gsea/msigdb/) and a gene set with interferon-stimulated genes (ISG) obtained from Wright et al. [36] (see Additional file 1: Table S8) were used. The GSEA test indicates the enrichment of gene sets in either upregulated or downregulated genes based on the fold changes contrasting 32D cells with BCR-ABL vs JAK2V617F, JAK2V617F vs EV, and BCR-ABL vs EV.

### Statistical analysis

Statistical analysis was performed with GraphPad Prism software 5.0 (GraphPad Software, La Jolla, CA, USA) and the two-tailed Student’s *t* test and one-way ANOVA (Dunnett’s test). Significant differences were defined as *p < 0.05, **p < 0.01, and ***p < 0.001. Mean and standard deviation (SD) are indicated. All experiments have been performed at least three times, if not indicated differently.

## Results

### JAK2V617F- and BCR-ABL-positive cells are differentially targeted by IFNa and show opposite effects on interferon-stimulated gene (ISG) expression

**Fig. 1:**
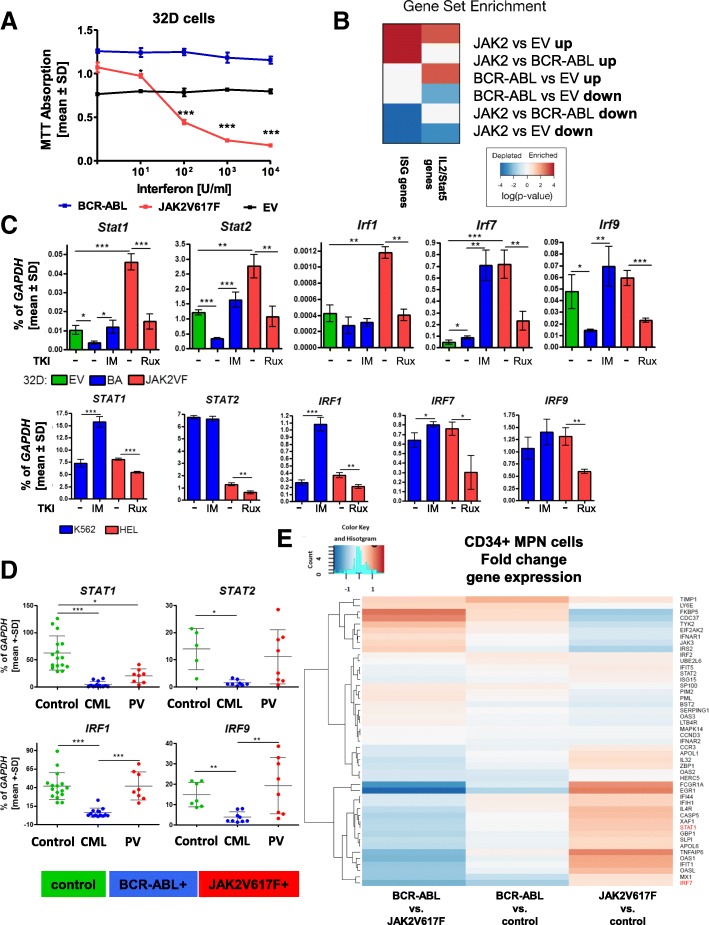
IFNa effects and mRNA regulation in 32D-BCR-ABL- and 32D-JAK2V617F-positive cells. 32D-JAK2V617F-positive cells showed concentration-dependent decreased viability after 72 h of IFNa treatment and activation of interferon target gene expression compared to BCR-ABL cells. **a** 32D-EV (black), 32D-BCR-ABL (blue), and 32D-JAK2V617F (red) were treated with IFNa (0–10^4^ U/ml) for 72 h, and the viability was measured by MTT. 32D-EV cells were grown in the presence of 5 ng/ml IL-3. **b** Gene set enrichment analysis of ISG and IL2/STAT5 genes. **c** Microarray results were validated by RT-qPCR. mRNA expression of EV-positive (green), BCR-ABL-positive (blue), or JAK2V617F-positive (red) cells was analyzed in murine 32D cells, which were WEHI starved for 24 h, or in human cell lines harboring the same genetic alteration. Oncogene-transduced cells were treated with 1 μM TKI (BCR-ABL + imatinib; JAK2V617F + ruxolitinib) for 18 h prior to analysis. The expression level was calculated as a percentage of *Gapdh*. Depicted are the mean values ± SD. *p < 0.05, **p < 0.01, ***p < 0.001. **d** Mononucleated cells were isolated from peripheral blood samples of healthy volunteers (green), BCR-ABL-positive (blue) CML patients, and JAK2V617F positive (red) PV patients, and the mRNA expression was analyzed as a percentage of *GAPDH* and analyzed with one-way ANOVA and Dunn’s multiple comparison test. *STAT1* and *IRF1*: controls: *n* = 16, CML: *n* = 13, PV: *n* = 8; *STAT2*: controls: *n* = 5, CML: *n* = 8, PV: *n* = 6. *IRF9*: controls: *n* = 7, CML: *n* = 9, PV: *n* = 6. **e** Gene expression microarray analysis of CD34-positive cells isolated from BCR-ABL-negative MPN or BCR-ABL-positive CML patients. Fold change of gene expression is shown, depicting downregulation of the analyzed gene in blue and upregulation in red

The effect of in vitro treatment with IFNa (0–10^4^ U/ml) on cell viability was analyzed in murine 32D cells, stably expressing BCR-ABL or JAK2V617F. 32D-EV (empty vector) cells served as maternal non-oncogene-expressing control, and cell viability was not altered by increasing IFNa concentrations (Fig. 1a). Viability of 32D-JAK2V617F cells was significantly reduced, while it remained essentially unchanged in 32D-BCR-ABL cells. Combination of IFNa with low doses of imatinib (IM 0.1 μM) sensitized 32D-BCR-ABL cells to IFNa and viability decreased in a concentration-dependent manner (Additional file 3: Figure S2). The viability of 32D-JAK2V617F cells was decreased to approximately 75% of control with 0.1 μM ruxolitinib alone (Rux), but there was no additive effect in combination with IFNa.

Gene expression profiling (GEP) of 32D-EV 32D-BCR-ABL, and 32D-JAK2V617F cells demonstrated that ISGs were differentially regulated by the two oncogenes (Additional file 4: Figure S3). This was not an unspecific effect, as illustrated by gene set enrichment analysis (GSEA), showing that IL2/STAT5 pathway-associated genes were similarly regulated by both oncogenes (Fig. 1b; Additional file 1: Table S8). Validation of *Stat1*, *Stat2*, *Irf1*, *Irf7*, and *Irf9* mRNA expression by RT-qPCR showed a significant downregulation of *Stat1*, *Stat2*, and *Irf9* in 32D-BCR-ABL cells in comparison to EV cells, while all five analyzed genes were significantly upregulated in 32D-JAK2V617F cells (Fig. 1c, upper panel). Imatinib and ruxolitinib reverted BCR-ABL- and JAK2V617F-induced changes, respectively (Fig. 1c, upper panel). These data were confirmed in human cell lines K562 (BCR-ABL-positive) and HEL (JAK2V617F-positive) (Fig. 1c, lower panel). *Stat2* expression was high in 32D-BCR-ABL cells and did not change upon imatinib treatment. Using 32D cell supernatants, we were able to exclude that the observed effects on ISG expression were due to soluble factors produced by JAK2V617F-positive cells, as collected supernatants were not capable to induce mRNA expression of the ISGF3 components (Additional file 5: Figure S4).

In order to analyze the impact of BCR-ABL and JAK2V617F on ISG mRNA expression in primary cells, peripheral blood samples from healthy donors, BCR-ABL-positive CML patients, and JAK2V617F-positive PV patients were tested. The expression of all analyzed genes was significantly reduced in the CML samples, while no significant changes were observed in the PV samples (Fig. 1d) except for *STAT1* expression, which was significantly reduced in PV samples but not to the same extent as in the CML samples. Importantly, *IRF1* and *IRF9* expression was significantly higher in JAK2V617F- vs. BCR-ABL-positive PBMNCs (Fig. 1d). IFNa responsiveness did not correlate with the ratio of bcr-abl/abl transcripts of the patients (data not shown). However, a significant correlation of JAK2V617F allele burden and ISG induction was observed for *IRF9* (*p* = 0.0492) (Additional file 6: Figure S5A), suggesting that *IRF9* expression reflects the malignant tumor burden in PV. The presence of additional mutations such as TET2 or ASXL1 mutations did not correlate with low expression of ISG genes (Additional file 6: Figure S5A). Importantly, GEP of CD34-positive cells of BCR-ABL-positive [34] and JAK2V617F-positive [37] MPN patients confirmed differential ISG expression patterns (Fig. 1e), observed in our 32D cell model system and the PBMCs, demonstrating that these changes were not simply due to different cell subpopulations or aberrant murine cell physiology.

### Combination treatment of IFNa and imatinib increases the IFNa response in BCR-ABL-positive cells, while ruxolitinib blocks IFNa-triggered signaling

**Fig. 2:**
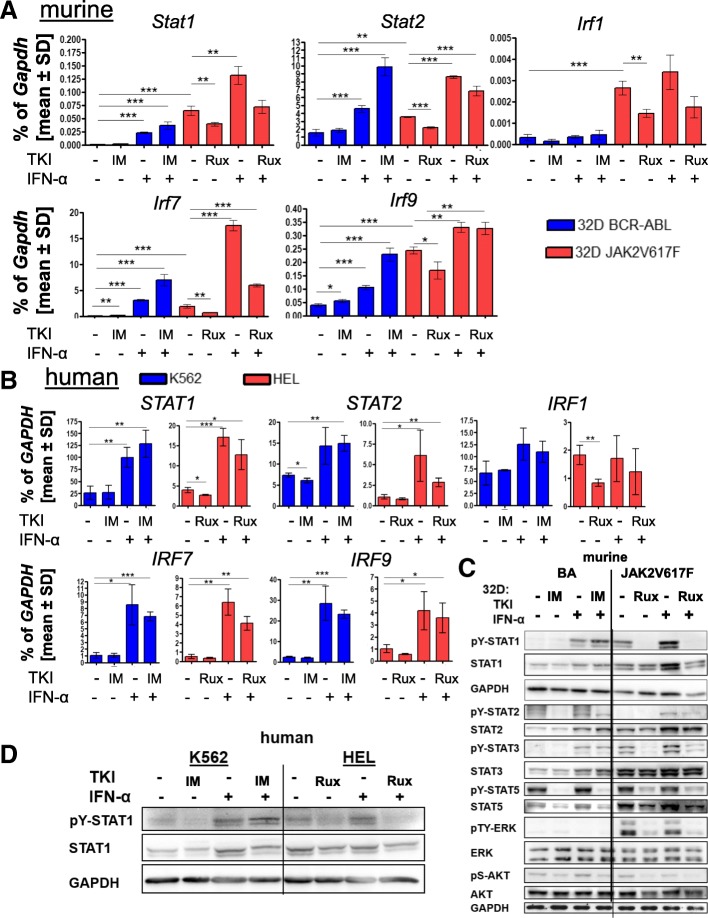
IFNa and TKI effects on mRNA and protein expression in cell lines and patient PBMCs. BCR-ABL-positive (blue) or JAK2V617F-positive (red) cells were treated with 1 μM imatinib (BCR-ABL) or ruxolitinib (JAK2V617F) or 100 U/ml IFNa or a combination of TKI and IFNa for 4 h. **a**, **b** mRNA expression of interferon target genes in transduced murine 32D cells or human K562 (BCR-ABL) or HEL (JAK2V617F) cells, shown as mean values ± SD as a percentage of *GAPDH* expression. *p < 0.05, **p < 0.01, ***p < 0.001. 32D cells were WEHI starved for 24 h before starting the experiment. **c** Western blot analysis of the canonical IFNa and oncogene-triggered signaling after TKI and/or IFNa treatment (4 h). The indicated antibodies were used, and GAPDH served as the loading control. **d** STAT1 expression and tyrosine phosphorylation (pY-STAT1) after 4 h of TKI and/or IFNa treatment in K562 and HEL cells

Next, we analyzed the effect of IFNa alone or in combination with either imatinib or ruxolitinib on mRNA expression of ISGs. Both 32D-BCR-ABL and 32D-JAK2V617F cells showed increased expression of *Stat1*, *Stat2*, *Irf7*, and *Irf9* after IFNa treatment (Fig. 2a). Moreover, IFNa-induced mRNA expression was further increased by the addition of imatinib in 32D-BCR-ABL cells, while the addition of ruxolitinib reduced IFNa-induced gene expression in 32D-JAK2V617F cells (Fig. 2a). Similar IFNa-induced effects were seen in the human cell lines K562 and HEL, but no additive effect of imatinib in K562 cells was observed (Fig. 2b).

When protein expression and tyrosine phosphorylation of major signaling molecules were analyzed in 32D-BCR-ABL and 32D-JAK2V617F cells after IFNa and/or TKI treatment, 32D-JAK2V617F cells showed higher STAT1 protein expression and phosphorylation compared to BCR-ABL-positive cells, and this phosphorylation was inhibited by ruxolitinib (Fig. 2c), suggesting that this was a JAK2V617F-mediated effect. In addition, 32D-EV cells showed STAT1 phosphorylation only after IFNa stimulation, comparable to BCR-ABL-positive cells (Additional file 6: Figure S5B). IFNa treatment increased total STAT1 protein expression and tyrosine phosphorylation in both BCR-ABL- and JAK2V617F-positive 32D cells and also in K562 and HEL cells (Fig. 2c, d). Interestingly, the combination of imatinib and IFNa further increased the phosphorylation of STAT1 in BCR-ABL-positive cells, while STAT1 phosphorylation was completely blocked in JAK2V617F-positive cells treated with ruxolitinib and IFNa (Fig. 2c, d). We observed differential constitutive activity of STAT2, STAT3, and ERK1/2 phosphorylation in 32D-BCR-ABL vs. 32D-JAK2V617F cells (Fig. 2c), with higher pY-STAT2 but lower pY-STAT3, and pTY-ERK1/2 levels in BCR-ABL- than JAK2V617F-positive cells. All of these were downregulated by the respective TKI, while IFNa-induced pY-STAT1 and pY-STAT3 were downregulated by ruxolitinib but not imatinib.

Thus, both the murine and human cell line results and the data gathered from the primary patient (PBMC and CD34+) cells demonstrated opposite effects of IFNa +/− TKI in JAK2V617F- vs. BCR-ABL-positive cells.

### CRISPR/Cas9-triggered knockout of STAT1 or STAT2 alters IFNa sensitivity of 32D-JAK2V617F cells

**Fig. 3:**
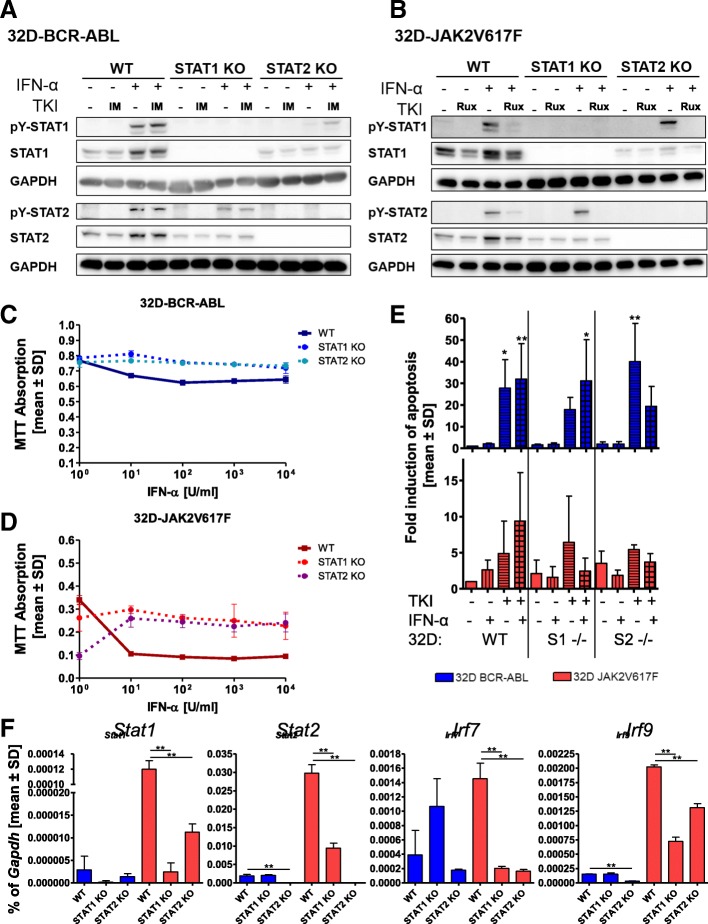
STAT1 or STAT2 knockout alters IFNa responsiveness only in JAK2V617F-positive cells. **a** 32D-BCR-ABL-WT, 32D-BCR-ABL-STAT1ko, and 32D-BCR-ABL-STAT2ko or **b** 32D-JAK2V617F-WT, 32D-JAK2V617F-STAT1ko, and 32D-JAK2V617F-STAT2ko cells were treated for 4 h with TKI (1 μM imatinib and ruxolitinib, respectively) or IFNa (100 U/ml) or a combination of both. SDS-Page and Western blotting were performed, and the indicated immunostainings were carried out. GAPDH served as the loading control. **c** 32D-BCR-ABL-WT, 32D-BCR-ABL-STAT1ko, and 32D-BCR-ABL-STAT2ko or **d** 32D-JAK2V617F-WT, 32D-JAK2V617F-STAT1ko, and 32D-JAK2V617F-STAT2ko cells were treated with increasing concentrations of IFNa, and cell viability was measured by MTT assay. **e** Indicated 32D cells were treated with TKI (0.5 μM imatinib and 0.1 μM ruxolitinib, respectively) or IFNa (100 U/ml) or a combination of both for 48 h, due to the rapid growth of untreated BCR-ABL-positive cells. PI staining was performed to discriminate between living and dead cells. Mean values ± SD are depicted. **f** Measurement of *Stat1*, *Stat2*, *Irf7*, and *Irf9* mRNA expression in the indicated cell lines. Expression was calculated as a percentage of *Gapdh*, and the mean values ± SD are depicted. *p < 0.05, **p < 0.01, ***p < 0.001. For all experiments, the respective 32D cells were WEHI starved for 24 h before starting the experiments

We generated *Stat1* and *Stat2* knockout 32D-BCR-ABL or 32D-JAK2V617F cells to better define the role of these two STAT proteins in the differential sensitivity to IFNa. Several knockout clones were generated with CRISPR/Cas9n technology, verified by Western blotting (Additional file 7: Figure S6), and one clone of each cell line was selected and treated with TKI, IFNa, or the combination (Fig. 3a, b). The results demonstrated that, in BCR-ABL-positive cells, genetic disruption of *Stat1* decreased Stat2 protein expression while preserving IFNa-induced STAT2 phosphorylation (Fig. 3a), did not alter viability of untreated cells (Fig. 3c), but abolished the already low IFNa response, with IFNa alone failing to induce apoptosis (Fig. 3c, e). There was no change in ISG expression in the absence of STAT1, suggesting that downregulation of these genes by BCR-ABL, as shown in Fig. 1, was STAT1-independent (Fig. 3f).

In JAK2V617F-positive cells, the absence of STAT1 led to a decrease of STAT2 protein while preserving IFNa-induced STAT2 phosphorylation and to an abrogation of most IFNa-induced effects (ISG expression, reduced cell viability, and with a tendency for induction of apoptosis), suggesting that both the JAK2V617F-induced sensitization to IFNa as well as IFNa-induced effects are STAT1-mediated (Fig. 3b, d–f, and Additional file 8: Figure S9a, b).

The absence of STAT2, in BCR-ABL-positive cells, led to a strong downregulation of STAT1 protein, including induction of pY-STAT1 by IFNa. *Irf9* gene expression was significantly downregulated in the absence of STAT2, and there was a trend towards decreased expression of other ISGs, but there was no effect on cell viability or apoptosis, regardless of the presence or absence of IFNa (Fig. 3a, c, e, f).

Conversely, upon STAT2 disruption, IFNa-induced STAT1 phosphorylation was preserved in JAK2V617F cells, with the latter showing nearly complete loss of STAT1 protein (Fig. 3a, b). Also, STAT2 knockout led to a significant reduction of all ISGs in JAK2V617F-positive cells, and these cells showed lower viability and a higher rate of apoptosis (Fig. 3d, e) than their STAT2 wt counterparts. However, IFNa increased viability and decreased, although not significantly, apoptosis in these cells (Fig. 3d, e), suggesting a role of STAT2 in promoting IFNa-induced apoptosis in JAK2V617F cells.

### Differential capacities of STAT2 in regulating STAT1 expression depending on the oncogenic background

For a better understanding of the role of unphosphorylated (referring to the regulatory tyrosines Y701 in STAT1 and Y689 in STAT2) STAT1/2 in a BCR-ABL or JAK2V617F background, 32D-BCR-ABL- and 32D-JAK2V617F-S1ko or 32D-JAK2V617F-S2ko cells were reconstituted with wt-STAT1, wt-STAT2, the dominant negative form STAT1Y701F (STAT1Y/F), or the phospho-deficient STAT2Y689F (STAT2Y/F) mutant, respectively.

**Fig. 4:**
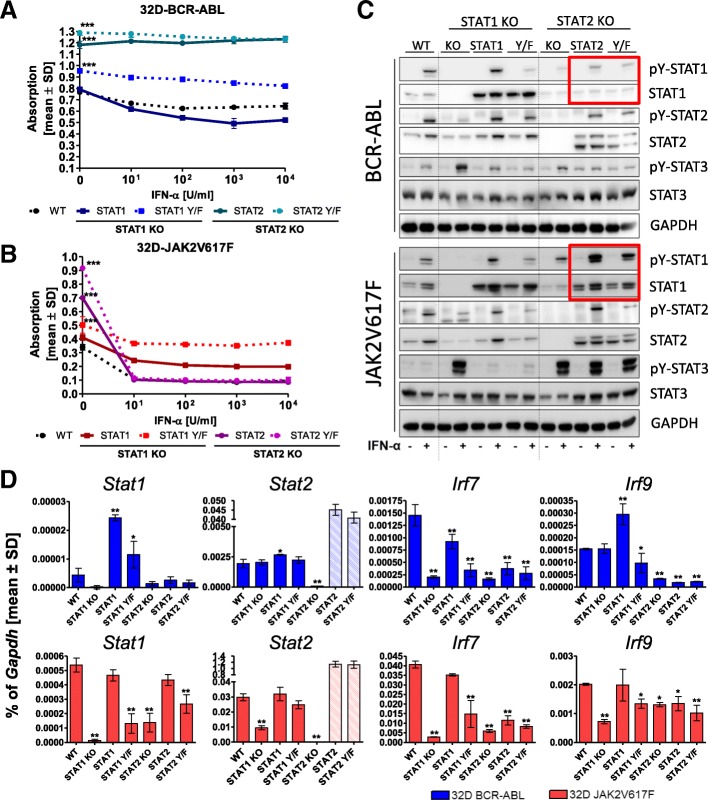
Reconstitution of 32D-BCR-ABL and 32D-JAK2V617F STAT1ko and STAT2ko cells. 32D-BCR-ABL and 32D-JAK2V617F cells (depicted as WT, respectively), which passed through the CRISPR STAT KO process but showed no knockout, were used as control cell lines. 32D-BCR-ABL (**a**) and 32D-JAK2V617F (**b**) STAT1ko or STAT2ko cells reconstituted with wt-STAT1, wt-STAT2, STAT1Y701F (Y/F), or STAT2Y689F (Y/F) were applied in a MTT assay and treated with the indicated concentrations of IFNa (0–10^4^ U/ml) for 72 h. MTT assays have been performed four times in independent experiments, and untreated controls were analyzed with one-way ANOVA and Dunn’s multiple comparison test. Further statistical analysis can be found in Additional file 9: Figure S7A, B. **c** Western blot analysis of the 32D cell lines depicted in **a** and **b** treated for 4 h with 100 U/ml IFNa or left untreated. Phosphorylation of STAT1, STAT2, and STAT3 was analyzed. GAPDH served as the loading control. **d** mRNA expression of interferon-stimulated genes in the indicated cell lines. *Stat2* qPCR primer detected the ectopically expressed *Stat2* mRNA, explaining the strong upregulation, and endogenous *Stat2* can thus not be evaluated in the reconstituted experiments. Gene expression was calculated as a percentage of *Gapdh*, and the mean values ± SD are depicted. *p < 0.05, **p < 0.01, ***p < 0.001. The respective 32D cells were grown in IL3-source-free medium 24 h before application into the experiment

We observed that STAT2 and STAT2Y/F expression in 32D-BCR-ABL- and 32D-JAK2V617F-S2ko cells led to increased viability in the absence of IFNa treatment (Fig. 4a, b). As expected, STAT1 reconstitution in BCR-ABL- and JAK2V617F-positive STAT1ko cells increased IFNa responsiveness (Fig. 4a, b and Additional file 9: Figure S7A, B). The IFNa response was only slightly reduced in 32D-JAK2V617F-S1ko STAT1Y/F-expressing cells, also shown in a titration of lower IFNa dosages (Fig. 4a, b and Additional file 9: Figure S7). Interestingly, 32D-JAK2V617F-S2ko-STAT2(Y/F) cells were highly responsive to IFNa treatment (Fig. 4b and Additional file 9: Figure S7), demonstrating that Y689 phosphorylation of STAT2 is not a prerequisite for IFNa efficacy.

In the following, the phosphorylation pattern of STAT1, STAT2, and STAT3 were analyzed to potentially explain the striking differences of STAT2(Y/F) reconstitution in BCR-ABL- and JAK2V617F-positive-S2ko cells. In 32D-BCR-ABL-S1ko cells stimulated with IFNa, STAT1 but also STAT1Y/F expression increased pY-STAT2 as well as the overall STAT2 protein level (Fig. 4c). Therefore, STAT1 but not its phosphorylation at Y701 is necessary for STAT2 expression in BCR-ABL-positive 32D cells. Meanwhile, STAT1Y/F expression in IFNa-stimulated 32D-JAK2V617F-S1ko cells failed to upregulate STAT2 to the same extent (Fig. 4c).

Re-expression of STAT2 as well as STAT2Y/F normalized or even increased endogenous STAT1 expression and phosphorylation in JAK2V617F-positive STAT2ko cells but not in BCR-ABL-positive cells (Fig. 4c, red rectangle). This upregulation in 32D-JAK2V617F-S2ko-STAT2(Y/F) cells could explain the enhanced IFNa sensitivity found in all of our assays (Figs. 1, 2, and 4b). IFNa-induced pY-STAT3 was enhanced by STAT1 disruption and decreased by reconstitution with STAT1 or STAT1Y/F in both cell lines, demonstrating that STAT1 and STAT3 are competing for the same phospho-binding sites at the IFN receptors (Fig. 4c). However, there was a marked difference of IFNa-induced STAT3 phosphorylation in BCR-ABL- and JAK2V617F-positive cells in the absence of STAT2: strong IFNa-induced pY-STAT3 persisted only in JAK2V617F- but not BCR-ABL-positive cells despite re-expression of STAT2 or the STAT2Y/F mutant (Fig. 4c). In general, STAT3 was only weakly phosphorylated in BCR-ABL vs. JAK2V617F expressing cells (Fig. 2c). Additional file 10: Figure S8 illustrates the differences in pY-STAT1 signaling in BCR-ABL- vs. JAK2V617F-positive cells, shown in Fig. 4c, at higher contrast. The Y/F mutants showed residual pY signals, most likely due to unspecific antibody binding, as the correct sequence of all mutants was confirmed by Sanger sequencing (data not shown).

In the following, ISG expression was further analyzed in reconstituted 32D-STATko cells (Fig. 4d). In JAK2V617F-positive cells, expression of all ISGs was fully rescued by STAT1 reconstitution and to a lesser degree by the STAT1Y/F mutant (Fig. 4d), demonstrating STAT1 as one of the key downstream effectors of JAK2V617F in stimulating the IFNa pathway. In BCR-ABL-positive cells, STAT1 reconstitution led to increased *Stat1* and *Irf9* expression, overriding BCR-ABL-mediated suppression of these genes (Fig. 4d). In the presence of IFNa, ISG expression in STAT1 or STAT2 ko cells was abrogated but strongly induced in STAT1- (and to a lesser extent, STAT1Y/F)-reconstituted BCR-ABL- and JAK2V617F-positive cells (Additional file 8: Figure S9A, B).

STAT2 reconstitution (and to a lesser extent, STAT2Y/F) was able to rescue ISG transcription (*Stat1* and *Irf7*, while *Irf9* was not decreased in the absence of STAT2) in JAK2V617F- but not BCR-ABL-positive cells (Fig. 4d). In all of these cells, reconstitution of STAT2 rescued IFNa-stimulated ISG expression at least partially (Additional file 8: Figure S9a, b).

Together, our results support the hypothesis that, in BCR-ABL-expressing cells, STAT2 is a survival factor but not an IFNa-sensitizing factor. In contrast, in JAK2V617F-positive cells, STAT2 is a survival as well as an IFNa-sensitizing factor.

### Histone modifications at TFBS in 32D-BCR-ABL and 32D-JAK2V617F cells correlate with gene expression

**Fig. 5:**
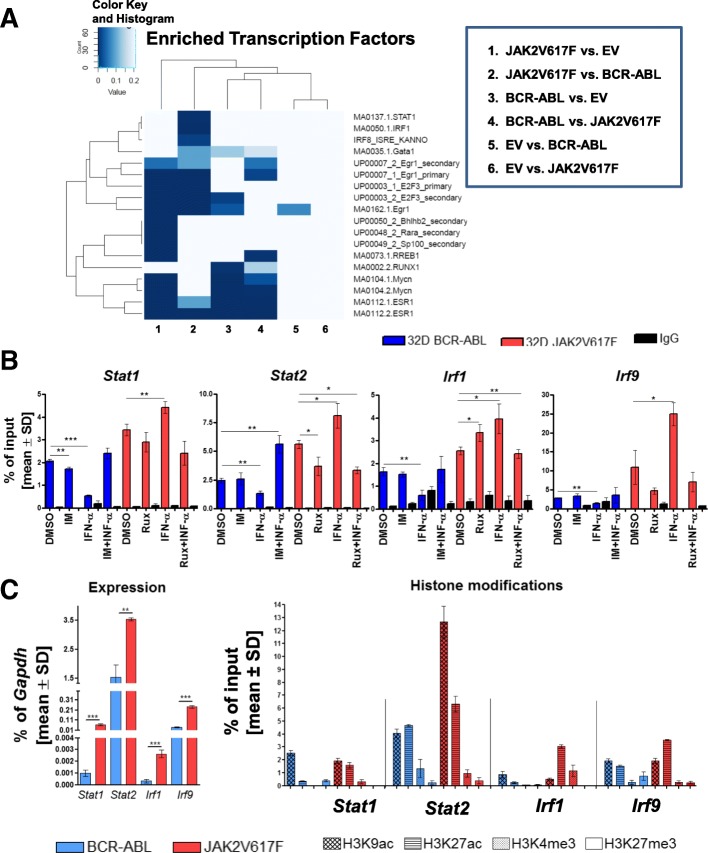
Histone modifications in the promoter region of interferon target genes differ between 32D-BCR-ABL- and 32D-JAK2V617F-positive cells. **a** Enrichment analysis of transcription factor binding sites (TFBS) in acetylation (H3K9ac) peaks based on ChIP-seq data. Differentially regulated genes were analyzed for the presence of TFBS in the acetylation peaks, and it was tested if the number of TFBS is significantly different. **b** 32D-BCR-ABL-transduced (blue) and 32D-JAK2V617F-transduced (red) cells were analyzed for changes of H3K9 acetylation in the promoter region of *Stat1*, *Stat2*, *Irf1*, and *Irf9* after 4 h of 1 μM TKI and/or 100 U/ml IFNa treatment by ChIP-PCR and were tested for differences in comparison to the corresponding DMSO-treated cell line. *p < 0.05, **p < 0.01, ***p < 0.001. **c** Further histone marks such as H3K27 acetylation (active mark), H3K4 tri-methylation (active mark), or H3K27 tri-methylation (repressive mark) were measured by ChIP-PCR (right side) and correlated with *Stat1*, *Stat2*, *Irf1*, and *Irf9* mRNA expression (left side) in these 32D-BCR-ABL (blue) and 32D-JAK2V617F (red) cells. Binding was calculated as the percentage of input and shown as mean value ± SD. ChIPs have been performed twice and expression analysis three times in triplicate in independent experiments. Oncogene-expressing 32D cell lines were growing without WEHI for the experimental procedure

Screening for differences in the acetylation profile in 32D-EV 32D-BCR-ABL, or 32D-JAK2V617F was done by ChIP-seq experiments to correlate these findings with the expression array data. ChIP-seq analysis revealed specific active regulatory regions (Additional file 11: Figure S10) and a significant enrichment of STAT1, IRF1, and IRF8 binding sites in the acetylated regions in 32D-JAK2V617F compared to 32D-BCR-ABL cells (Fig. 5a; Additional file 1: Table S9).

Based on these findings, validation of H3K9 acetylation (H3K9ac) by ChIP-PCR was performed at the promoter of ISGs at ISRE sites after TKI and/or IFNa treatment. *Stat1*, *Stat2*, *Irf1*, and *Irf9* showed significantly higher H3K9ac in 32D-JAK2V617F cells compared to 32D-BCR-ABL cells (Fig. 5b). Interestingly, IFNa reduced the acetylation at the promoter of *Stat1*, *Stat2*, *Irf1*, and *Irf9* in 32D-BCR-ABL cells, but increased it in all four promoter regions in 32D-JAK2V617F cells (Fig. 5b). No changes were observed when 32D-BCR-ABL cells were treated with imatinib alone or in combination with IFNa, except for induction of *Stat2* promoter acetylation with the combination treatment. Ruxolitinib treatment inhibited IFNa-induced H3K9ac of all four gene promoter regions in 32D-JAK2V617F cells (Fig. 5b).

However, since IFNa induces high mRNA expression (Fig. 2a) despite lower H3K9ac levels (Fig. 5b) in 32D-BCR-ABL cells, further mechanisms of gene regulation were likely to play a role. Thus, further histone marks were analyzed and compared to ISG mRNA expression patterns (Fig. 5c). The results confirmed close correlation of high mRNA expression with a low abundance of the repressive H3K27me3 chromatin mark but high abundance of active H3K9ac, H3K27ac, and H3K4me3 marks (Fig. 5c).

## Discussion

Since its discovery almost 60 years ago, IFNa and its pegylated form, PegIFNa, have been successfully used therapeutically in various malignancies, including MPN [33]. Nevertheless, the IFNa response is dependent on the MPN subtype, with IFNa monotherapy inducing complete hematologic remission (CHR) rates of 76% and complete molecular remission rates of 18% in Ph neg MPNs such as PV or ET [38], but no more than 55% CHR rates in CML [39, 40] and much lower rates in overt PMF [41, 42].

In this study, we compared the IFNa response of BCR-ABL- vs. JAK2V617F-positive cells and their dependence on the transcription factors STAT1 and STAT2. BCR-ABL cells were resistant to growth inhibition and apoptosis by IFNa single treatment, while 32D-JAK2V617F cells were highly sensitive (Fig. 1a). It has been described that BCR-ABL suppresses IFN-inducible gene expression and IFNa responsiveness in Ba/F3-BCR-ABL cells [20]. We confirmed these results and significantly extended the analysis to human cell lines, PBMCs, and CD34+ cells from BCR-ABL-positive vs. BCR-ABL-negative MPN patients (Figs. 1 and 2). Although CML PBMCs show clear reduction of the analyzed ISGs, supporting our cell line data, the regulation in PV-derived PBMC samples was heterogeneous. This might be explained by the differing allelic burden in PBMC in comparison to 100% positivity in cell lines. ISG suppression by BCR-ABL may be partially explained by lysosomal degradation of IFNAR1 in BCR-ABL-expressing cells, resulting in reduced IFNa sensitivity [26] and the presence of pY-STAT2, reported to be involved in ISG repression [43].

Using our CRISPR/Cas9n-mediated STAT1 and STAT2 knockout cell lines, we were able to demonstrate that, in BCR-ABL-positive cells, STAT2 is essential for IFNa-induced STAT1 phosphorylation (Fig. 3a), supported by the observation that pY-STAT2 recruits STAT1 to the activated IFNa receptor [44]. Importantly, we demonstrate that, in JAK2V617F-expressing cells, STAT1 phosphorylation by IFNa stimulation is independent from STAT2 (Fig. 3b), suggesting that receptor recruitment is different in JAK2V617F-positive cells. Furthermore, STAT2ko is less effective in repression of ISG expression (Fig. 3f and Additional file 10: Figure S8A, B) in comparison to JAK2V617F-STAT1ko cells, presumably due to the retained pY-STAT1 after IFNa stimulation (Fig. 3b).

**Fig. 6:**
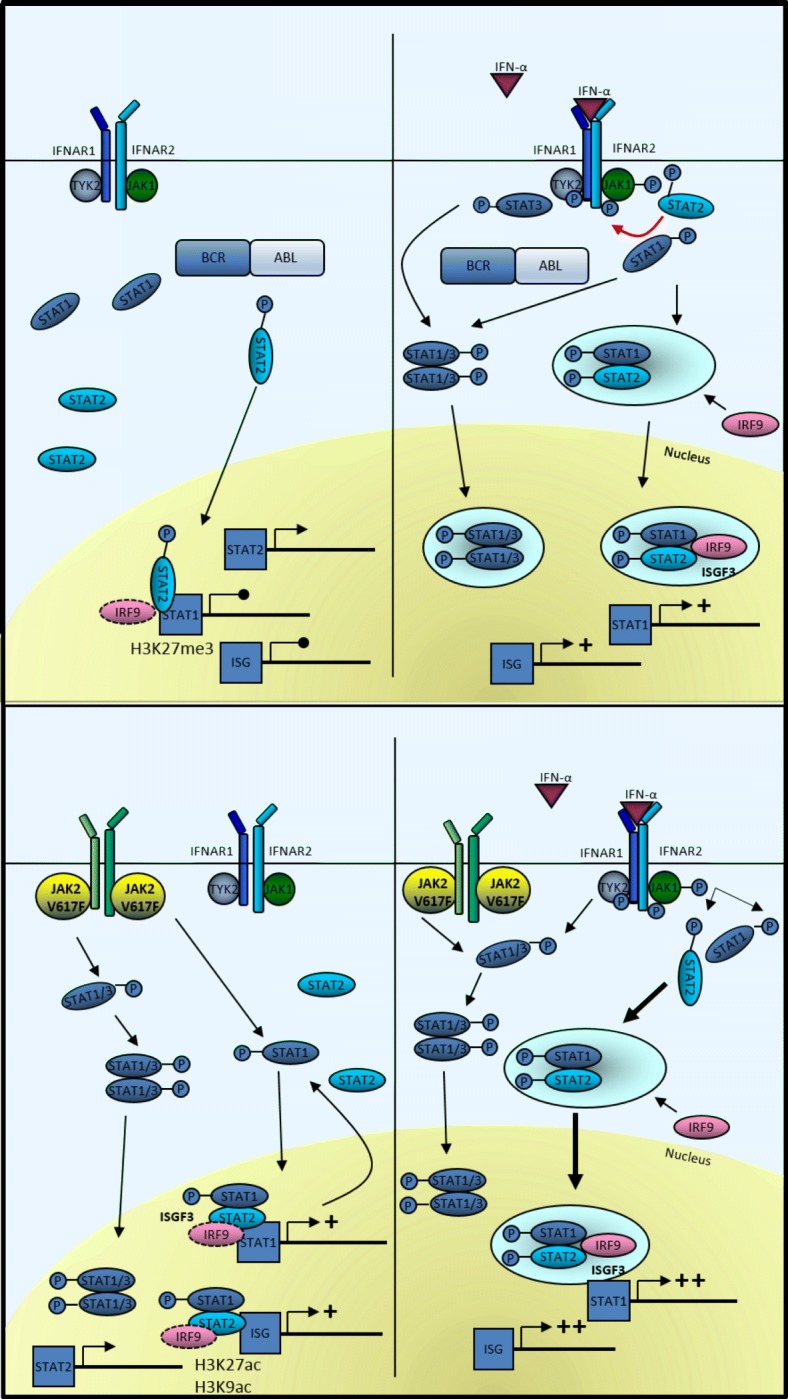
Simplified overview of ISG regulation by STAT1 and STAT2 in BCR-ABL- and JAK2V617F-positive cells. In BCR-ABL-expressing cells, STAT2 is partially phosphorylated leading to ISG repression [43] and is not capable to induce STAT1 expression. In addition, chromatin marks negatively regulating gene expression are present (i.e., H3K27me3). STAT2, although not phosphorylated in JAK2V617F-positive cells, can induce STAT1 expression in the presence of histone marks representing active promoters (i.e., H3K9ac and H3K27ac). Upon stimulation with IFNa, STAT2 is essential for STAT1 phosphorylation at its tyrosine residue Y701 in BCR-ABL-positive cells (indicated by red arrow). The ISGF3 complex is formed and ISG expression is induced. IFNa stimulation of JAK2V617F-positive cells leads to ISGF3 complex formation and ISG as well as STAT1 promoter access. The equilibrium of STAT1/STAT1 and STAT1/STAT3 dimers shifts in dependence of the amount of active STAT2. STAT1/3 illustrates different dimer options: STAT1/STAT1, STAT1/STAT3, and STAT3/STAT3. In BCR-ABL-expressing cells, STAT3 is phosphorylated after IFNa binding to its receptor. P, tyrosine phosphorylation

In the following, we demonstrate that STAT2Y689F induces STAT1 expression in JAK2V617F-positive cells but not in BCR-ABL-expressing cells (Fig. 4c and Fig. 6). We found higher levels of the negatively regulating histone mark H3K27me3 [45] in BCR-ABL-expressing cells, probably explaining the incapacity to upregulate STAT1 expression. Furthermore, we demonstrated a re-induction of ISGs in BCR-ABL-positive cells after imatinib treatment and a further increase when cells were treated with a combination of TKI and IFNa (Fig. 2), most likely due to positive feedback of re-induced STAT1, loss of negatively regulating histone marks, and rescue of IFNAR1 surface expression after pretreatment of CML cell lines with TKI [26].

Previous reports assessing the mechanism of IFNa resistance in CML patients found both STAT1 deficiency [17] and SOCS3 overexpression [18] to play an important role. However, SOCS3 overexpression was only detected at the blast crisis but not chronic phase stage of CML [18]. Furthermore, while STAT1 expression was well correlated with the in vivo IFNa response in one study [17], BCR-ABL was only found to inhibit STAT1 phosphorylation but not STAT1 protein expression in another study [20]. Indeed, overexpression of STAT1 in our 32D-BCR-ABL cells was only marginally capable to induce IFNa efficacy, both in the presence or absence of endogenous STAT1 (Fig. 4 and Additional file 7: Figure S6). However, STAT2 reconstitution in STAT2-deficient BCR-ABL- and JAK2V617F-positive cells increased basal cell viability but did not change upon IFNa treatment (Fig. 4a). It needs to be mentioned that ectopic expression of STAT2 did not necessarily rescue the observed results in STAT2 +/+ cells. Ectopic expression of STAT2 is probably too high, which would suggest high cellular sensitivity to changes in STAT2 expression.

These results suggest that the ratio of different STAT proteins is crucial for cell proliferation, survival, and IFNa responsiveness in CML. In addition to differential STAT1/STAT2 signaling in the clonal cells themselves, cell-extrinsic effects such as IFNa-mediated activation of the immune system is certainly important [46]. However, in the present study, we specifically focused on the cell-autonomous effects of IFNa.

Contribution of STAT proteins in IFN signaling is very complex as most STATs can form homo- as well as hetero-dimers in phosphorylated and unphosphorylated state, thereby activating distinct subsets of genes [47, 48]. The expression level of STAT3 and its phosphorylation is crucial for the STAT1 transcriptional profile, as higher (phospho)-STAT3 levels reduce the amount of STAT1 homodimers and at the same time support the ISGF3-dependent gene expression [49], thereby influencing IFNa responsiveness. STAT3 was only phosphorylated in 32D-JAK2V617F cells but not in BCR-ABL-expressing cells (Fig. 2c), therefore supporting ISGF3 complex formation. Surprisingly, 32D-JAK2V617F-S1ko-STAT1Y/F cells were still responsive to IFNa (Fig. 4b and Additional file 10: Figure S8), suggesting the ISGF3 complex formation even without phosphorylation of STAT1 at its regulatory tyrosine 701.

Re-expression of STAT2 and STAT2YF led to increased viability of BCR-ABL- and JAK2V617F-positive S2ko cells. Nevertheless, only JAK2V617F-S2ko-STAT2(Y/F) cells were sensitized to low doses of IFNa, presumably due to upregulation of STAT1 protein expression (Fig. 4a, b). At the same time, while targeted disruption of STAT2 reduced basal viability of JAK2V617F cells, IFNa treatment increased cell viability, possibly through hyper-activation of STAT3 and reduced negative regulation (Figs. 3d and 4c). Intriguingly, ectopic expression of STAT2(Y/F) did not decrease pY-STAT3 to normal levels in 32D-JAK2V617F-S2ko cells (Fig. 4c), which was in line with only partial reconstitution of ISG induction (Additional file 8: Figure S9a and b) [50] and suggested that JAK2V617F but not BCR-ABL alters the requirement for STAT2 in the response to IFNa. These results argue for a differential and significant role of STAT2 but also STAT3 in BCR-ABL- vs. JAK2V617F-expressing cells (Fig. 6). In addition, our data also indicate a leading role for pY-STAT1 and STAT2, as a regulator of STAT1 transcription, in JAK2V617F-expressing cells for induction of apoptosis.

STAT binding at the promoter of ISGs is strongly influenced by histone modifications altering promoter accessibility. In former studies, it was demonstrated that histone acetylation is important for hematopoietic differentiation, which can be counteracted by overexpression of HDAC1 [51]. Our H3K9ac ChIP-seq data revealed an increase of acetylation at the binding sites of interferon-regulated transcription factors in line with an increase of IFNa target genes in JAK2V617F-positive cells, providing a link between acetylation and active gene transcription. In addition, STAT1 and STAT2 have been associated with histone acetyltransferases, resulting in localized acetylation of histones [52, 53]. Our data lead to the conclusion that acetylation at the promoter of ISGs in 32D-JAK2V617F but not 32D-BCR-ABL cells can be correlated with gene expression, with or without a response to TKI and IFNa treatment. HDAC inhibitors showed selective inhibition of ISGs that are activated by STAT1 and STAT2 [54]. In addition, IRF9 was shown to be important for the binding of RNA polymerase II to the promoter of these target genes to modulate the HDAC function and gene expression. Analysis of additional histone modifications confirmed a complex time and cell type-specific mechanism of gene regulation, as the presence of different modifications correlated with mRNA levels in the analyzed cells (Fig. 5c).

## Conclusions

Taken together, in BCR-ABL-positive cells, STAT2 was found to be a survival factor but not an IFNa-sensitizing factor, indicating an important role of STAT2 protein in the prediction of responsiveness of CML patients to IFNa therapy. In contrast, in JAK2V617F-positive cells, STAT2 is a survival and an IFNa-sensitizing factor, regulating STAT1 expression even in its unphosphorylated state (Fig. 6). Therefore, the complex network of STAT1, STAT2, and STAT3 in their unphosphorylated and phosphorylated states could probably help to predict the response to IFNa. Moreover, our data suggest that the analysis of the composition of histone modifications may be useful for the prediction of gene expression and response to therapy.

## Additional files

Additional file 1: Supplementary Material & Methods. **Table S1.** Primer for cloning STAT1 and STAT2 with included restriction sites. **Table S2.** STAT1 and STAT2 primers for mutagenesis. **Table S3.** RT-qPCR primer for gene expression analysis. **Table S4.** Western blot antibodies. **Table S5.** gRNA Oligonucleotides. **Table S6.** ChIP antibodies. **Table S7.** ChIP-PCR Primer. **Table S8.** GSEA of a set of 45 interferon-stimulated genes (ISG). **Table S9.** Statistics for the enrichment analysis of transcription factor binding sites (TFBS) in acetylation (H3K9ac) peaks. (DOCX 44 kb)

Additional file 2: **Figure S1.** Knockout of STAT1 or STAT2 with CRISPR/Cas9n technology. Four guide RNAs have been generated for STAT1 or STAT2 knockout in 32D-BCR-ABL and 32D-JAK2V617F cells, respectively. Excised exons are given. (PDF 19 kb)

Additional file 3: **Figure S2.** MTT assay of 32D-BCR-ABL and 32D-JAK2V617F cells treated with IFNa. 32D-BCR-ABL-(blue) and 32D-JAK2V617F-(red) positive cells were treated with IFNa (0–10^4^ U/ml) alone (continuous lines) or in combination with 0.1 μM imatinib (IM) or ruxolitinib (Rux) (dotted lines) for 72 h and the viability was measured by MTT. Viability was normalized to the untreated control and mean values ± SD are depicted. The respective 32D cells were WEHI starved for 24 h before starting the experiments. Experiments were performed in triplicate and conducted three times. (PDF 27 kb)

Additional file 4: **Figure S3.** BCR-ABL reduces ISG expression in 32D cells. Gene expression microarray analysis of 32D-EV, 32D-BCR-ABL, or 32D-JAK2V617F cells. Fold change of gene expression is shown, depicting downregulation of the analyzed gene in blue and upregulation in red. (PDF 134 kb)

Additional file 5: **Figure S4.** Effect of extrinsic soluble factors on gene expression in 32D-EV- or 32D-JAK2V617F-positive cells. Supernatant of WEHI-starved 32D-EV- or 32D-JAK2V617F-positive cells was generated overnight, and after removal of the cells, fresh EV (green) or JAK2V617F-(red) positive cells were incubated with the supernatant for 2 h prior to RNA extraction to analyze the expression of IFN target genes. Mean ± SD values are shown as % of *Gapdh.* Independent experiments were performed three times and in triplicate, respectively. (PDF 25 kb)

Additional file 6: **Figure S5.** Correlation of ISG expression and JAK2V617F allelic burden and Western blot of 32D EV, BCR-ABL, or JAK2V617F cells. A, ISG expression (% of *GAPDH*) and JAK2V617F allelic burden (in %) were plotted against each other and a significant correlation was only found for IRF9 (*p* = 0.0492). Samples with additional mutations (*TET2*, *ASXL1*, or *EZH2*) were highlighted with a red circle. B, Western blot analysis of pY-STAT1 and overall STAT1 protein after TKI and/or IFNa treatment (4 h) of 32D EV (only −/+ IFNa), BCR-ABL, or JAK2V617F cells. All cell lines were starved of WEHI (source of IL-3) for 24 h before treatment. *GAPDH* served as the loading control. The same Western blot is shown in Fig. 2c lacking 32D EV cells. (PDF 74 kb)

Additional file 7: **Figure S6.** Confirmation of successful STAT1 or STAT2 knockout. Western blotting of several 32D-BCR-ABL or 32D-JAK2V617F STAT1 or STAT2 knockout clones. STAT2 antibody was used to confirm the knockout, and GAPDH served as the loading control. 32D cells were WEHI starved for 24 h before starting the experiment. wt – wild-type clones, ko – knockout clones, het – presumed heterozygous clones (PDF 134 kb)

Additional file 8: **Figure S9.** Full RT-qPCR panels of tested ISGs. Illustration of the RT-qPCR results of 32D-BCR-ABL- and 32D-JAK2V617F-WT or -STATko or -STAT1(Y/F) and STAT2(Y/F) reconstituted cell clones treated with IFNa (100 U/ml) or left untreated (triplicate), corresponding to the data given in Figs. 3f and 4d. (a) *Stat1* and *Stat2*, (b) *Irf7* and *Irf9*. Stat2 qPCR primer detected the ectopically expressed *Stat2* mRNA, explaining the strong upregulation, and endogenous *Stat2* can thus not be evaluated in the reconstituted experiments (gray bars). Independent experiments were performed three times. (PDF 56 kb)

Additional file 9: **Figure S7.** Comparison of CRISPR/Cas9 manipulated 32D cell lines treated with 100 U IFNa in survival and titration of lower IFNa dosages. Indicated (A) 32D-BCR-ABL and (B) 32D-JAK2V617F cell lines were analyzed in an MTT assay and treated with 100 U IFNa for 72 h (abstracted from Fig. 4a, b). Absorption was normalized to untreated control cells and statistically analyzed using a *t* test. Mean values ± SD are indicated. *p < 0.05, **p < 0.01, ***p < 0.001. C, 32D-JAK2V617F cells, -S1ko and S2ko cells, reconstituted with STAT1 (S1RE), STAT1Y701F (S1YF), STAT2 (S2RE), or STAT2Y689F were treated with IFNa (0, 1, 5, 10 and 100 U/ml) for 72 h, and the viability was measured by MTT. Mean values ± SD are depicted. Independent experiments were performed three times and in triplicate, respectively. The respective 32D cells were WEHI starved for 24 h before starting the experiments. (PDF 26 kb)

Additional file 10: **Figure S8.** Illustration of pY-STAT1 in 32D-JAK2V617F cells in the absence of IFNa. STAT1 and pY-STAT1 stainings of Fig. 4c, shown with increased contrast. The arrows are indicating the pY-STAT1 bands in 32D-BCR-ABL- and 32D-JAK2V617F-STATko cells. (PDF 203 kb)

Additional file 11: **Figure S10.** H3K9 acetylation profile at ISGs. The ChIP-seq acetylation was visualized with the Integrative Genomics Viewer (IGV). Shown are the H3K9 acetylation peaks at the genomic regions of *Irf1*, *Irf17*, *Irf9*, and *Stat1* in 32D-JAK2V7F (JAK2V617F) (red), 32D-BCR-ABL (blue), and 32D-EV (green). (PDF 108 kb)

## Abbreviations

ChIP: Chromatin immunoprecipitation
CHR: Complete hematologic remission
CML: Chronic myeloid leukemia
ET: Essential thrombocythemia
EV: Empty vector
GEP: Gene expression profiling
GSEA: Gene enrichment analysis
IFNa: Interferon alpha
IFNAR: Interferon alpha receptor
IM: Imatinib
IRF: Interferon responsive factor
ISG: IFN-stimulated genes
ISGF3: IFN-stimulated gene factor 3
ISRE: IFN-stimulated response element
JAK: Janus kinase
KO: Knockout
MPN: Myeloproliferative neoplasms
PBMC: Peripheral blood mononuclear cells
pegIFNa: Pegylated IFNa
PMF: Primary myelofibrosis
PV: Polycythemia vera
Rux: Ruxolitinib
SD: Standard deviation
SOCS: Suppressor of cytokine signaling
STAT: Signal transducer and activator of transcription
TKI: Tyrosine kinase inhibitor
