# Supplementary material for: Differential roles of STAT1 and STAT2 in the sensitivity of JAK2V617F- vs. BCR-ABL-positive cells to interferon alpha
Source: J Hematol Oncol. 2019 Apr 2;12:36. doi: 10.1186/s13045-019-0722-9 (PMC6444528; doi:10.1186/s13045-019-0722-9)
Supplement: Supplementary file 1 — Supplementary Material & Methods. Table S1. Primer for cloning STAT1 and STAT2 with included restriction sites. Table S2. STAT1 and STAT2 primers for mutagenesis. Table S3. RT-qPCR primer for gene expression analysis. Table S4. Western blot antibodies. Table S5. gRNA Oligonucleotides. Table S6. ChIP antibodies. Table S7. ChIP-PCR Primer. Table S8. GSEA of a set of 45 interferon-stimulated genes (ISG). Table S9. Statistics for the enrichment analysis of transcription factor binding sites (TFBS) in acetylation (H3K9ac) peaks. (DOCX 44 kb) [file 13045_2019_722_MOESM1_ESM.docx]

**Differential Roles of STAT1 and STAT2 in the Sensitivity of JAK2V617F- vs. BCR-ABL-positive Cells to Interferon Alpha**

Claudia Schubert^1^, Manuel Allhoff^2^, Stefan Tillmann^1^, Tiago Maié^2^, Ivan G. Costa^2^, Daniel B. Lipka^3^, Mirle Schemionek^1^, Kristina Feldberg^1^, Julian Baumeister^1^, Tim H. Brümmendorf^1^, Nicolas Chatain^1,*^, and Steffen Koschmieder^1,*^

*^1^Department of Hematology, Oncology, Hemostaseology, and Stem Cell Transplantation, Faculty of Medicine, RWTH Aachen University, Aachen, Germany; ^2^ Institute for Computational Genomics, Faculty of Medicine, RWTH Aachen University, Aachen, Germany; ^3^Regulation of Cellular Differentiation Group, Division of Epigenomics and Cancer Risk Factors, German Cancer Research Center (DKFZ), Heidelberg , Germany; ^*^ These authors contributed equally to this work*

**Corresponding Author:**

Prof. Dr. med. Steffen Koschmieder, Department of Hematology, Oncology, Hemostaseology, and Stem Cell Transplantation, Faculty of Medicine, RWTH Aachen University, Pauwelsstr. 30, D- 52074 Aachen, Germany, Phone: +49-241-8036102, Fax: +49-241-8082449, E-mail: skoschmieder@ukaachen.de

Running Title: Differential IFNa-induced STAT1/STAT2 in CML vs PV

**Additional file 11: Supplementary Material & Methods**

**Methods**

*Collection of supernatants for the analysis of ISG upregulation by extrinsic factors*

Supernatant of 32D-EV and –JAK2V617F cells was obtained from overnight cultures. After centrifugation, the supernatant was filtered to remove residual cells. Analysis of the soluble factors was done by incubation of 32D-EV or –JAK2V617F cells in both supernatants for two hours, prior to RNA extraction.

Chromatin immunoprecipitation library preparation

ChIP sequencing libraries were prepared using the NEBNext Ultra DNA Library Prep Kit for Illumina (E7370, NEB) and the NEBNext Multiplex-Oligos for Illumina (E7335, NEB) following the manufacturer’s protocol. The adaptors were diluted 1:10 in sterile water before ligation. Cleanup of adaptor-ligated DNA was performed using a 1:1 ratio of SPRI beads and DNA. PCR amplification of libraries was performed on a LightCycler 480 (Roche) after addition of 0.5 µl of 100x SYBR Green using the following cycling conditions: initial denaturation (98°C, 30 sec) followed by 9-12 cycles (98°C, 10 sec; 65°C, 30 sec; 72°C, 30 sec). The PCR reactions were stopped, when the amplification curves had reached a fluorescence intensity of about 10. The PCR reactions were purified using SPRI beads at a ratio of 1:1 (beads:DNA). Sequencing libraries were multiplexed and subjected to 50 bp single-end sequencing on an Illumina HiSeq 2000 instrument (Illumina Inc., San Diego).

*Bioinformatic analyses*

Analysis of expression arrays was based on Bioconductor and analysis of ChIP-seq data was based on differential peak caller THOR (1)^37^. Detailed information is included in the Supplementary Material.

Bioinformatic Analyses

Gene expression values were normalized with the RMA package from Bioconductor (www.bioconductor.org). We kept all genes with log2 fold change > 1 and corrected p-value < 0.05 in at least of the compared conditions (BCR-ABL vs JAK2V617F, BCR-ABL vs EV and JAK2V617F vs EV). ChIP-seq libraries were aligned to mouse genome (mm9) using bwa^36^. Next, we used THOR to detect peaks gained/lost between previously described pairs of conditions. In the following, we selected all differential peaks with a corrected p-value < 0.05. THOR did not detect EV specific peaks in the comparison with JAK2V617F cells. Next, we used the MotifAnalysis tool to evaluate the enrichment of transcription factor binding sites (TFBS) inside differential peaks (www.regulatory-genomics.org). First, we performed motif match with a false discovery rate of 0.0001 using motifs from JASPAR (2) and UNIPROBE databases (3). We then applied a Fisher exact test to evaluate if a particular transcription factor had a higher proportion of binding inside differential peaks than in random genomic regions. We consider all differential expressed factors with an enrichment adjusted p-value < 0.05 in at least one set of differential peaks. Visualization of ChIP-seq profiles were performed with the integrative genomics viewer (IGV) (4).

1. Allhoff M, Sere K, J FP, Zenke M, I GC. Differential peak calling of ChIP-seq signals with replicates with THOR. Nucleic acids research. 2016;44(20):e153.

2. Mathelier A, Zhao X, Zhang AW, Parcy F, Worsley-Hunt R, Arenillas DJ, et al. JASPAR 2014: an extensively expanded and updated open-access database of transcription factor binding profiles. Nucleic acids research. 2014;42(Database issue):D142-7.

3. Newburger DE, Bulyk ML. UniPROBE: an online database of protein binding microarray data on protein-DNA interactions. Nucleic acids research. 2009;37(Database issue):D77-82.

4. Robinson JT, Thorvaldsdottir H, Winckler W, Guttman M, Lander ES, Getz G, et al. Integrative genomics viewer. Nature biotechnology. 2011;29(1):24-6.

Supplementary Tables

Table S1: Primer for cloning STAT1 and STAT2 with included restriction sites

| Target | Forward (5’ 🡪 3’) | Reverse (5’ 🡪 3’) |
| --- | --- | --- |
| *Stat1* | CATCCTCGAGATGTCACAGTGGTTCGAGCTTC | GACAGTATGATGAGCACAGTATAAGTTAACCATC |
| *Stat2* | CATCCTCGAGATGGCGCAGTGGGAGATGTTG | GGGATGGACTCTTGATACCTTCTGAGAATTCCATCGATT  Restriction site*: XhoI, HpaI, EcoRI* |

Table S2: STAT1 and STAT2 primers for mutagenesis

| Target gene | Forward primer (5’🡪3’) | Reverse primer (5’🡪3’) |
| --- | --- | --- |
| STAT1-Y701F | GACCCTAAGCGAACTGGATTCATCA  AGACTGAGTTG | CAACTCAGTCTTGATGAATCCAGTT  CGCTTAGGGTC |
| STAT2-Y689F | ACAGAGGAAATATTTGAAACATAAAC | TCTTCAAAATTAACTTTTTCCTGG |

Table S3: RT-qPCR primer for gene expression analysis

Murine primer pairs

| Target gene | Forward primer (5’🡪3’) | Reverse primer (5’🡪3’) |
| --- | --- | --- |
| *Stat1* | GATCGCTTGCCCAACTCTTG | ACTGTGACATCCTTGGGCTG |
| *Stat2* | GGCAGCGAATCACTCAAAGC | CACCAGAGTCAAGAAGCCGA |
| *Irf1* | TTGACAGCCCTCGAGGAAAC | CGACGCATGTCAATGCTCAG |
| *Irf7* | GGAGCTTGGATCTACTGTGGG | ATGCTGCATAGGGTTCCTCG |
| *Irf9* | CTGCTAAGCCTGGGACCTTT | GGCCATCCTTCTCCTTACCATAC |
| *Gapdh* | CCAATACGGCCAAATCC | CCAATACGGCCAAATCCG |

Human primer pairs

| Target gene | Forward primer (5’🡪3’) | Reverse primer (5’🡪3’) |
| --- | --- | --- |
| *STAT1* | TGTATGCCATCCTCGAGAGC | AGACATCCTGCCACCTTGTG |
| *STAT2* | CCGGGACATTCAGCCCTTTT | CTCATGTTGCTGGCTCTCCA |
| *IRF1* | GCCATTCACACAGGCCGATA | GTGGAAGCATCCGGTACACT |
| *IRF7* | TGTGCTGGCGAGAAGGC | TGGAGTCCAGCATGTGTGTG |
| *IRF9* | TTCTTCAAGGCCTGGGCAAT | CCTGGTGGCAGCAACTGATA |
| *GAPDH* | GAAGGTGAAGGTCGGAGT | GAAGATGGTGATGGGATTT |

Table S4: Western blot antibodies

| Antibody | Company |
| --- | --- |
| polyclonal anti-rabbit phospho-AKT (Ser473) (#9271L) | Cell signaling, Frankfurt, Germany |
| polyclonal anti-rabbit p-p44/42 MAPK (ERK1/2) (Thr202/Tyr204) (#9101S) |  |
| polyclonal anti-rabbit p44/42 MAPK (#9102S) |  |
| polyclonal anti-mouse/human phospho-STAT1 (Tyr 701) (#7649L) |  |
| polyclonal anti-mouse/human STAT1 (#9172S) |  |
| polyclonal anti-mouse/human phospho-STAT3 (Tyr 705) (#9131S) |  |
| polyclonal anti-mouse/human STAT3 (#12640S) |  |
| polyclonal anti-rabbit phospho-STAT5A/B (Tyr694/699) (#9351S) |  |
| polyclonal anti-rabbit STAT5 (#9363S) |  |
| Polyclonal anti-rabbit phospho-STAT2 (Tyr690) (#4441S) |  |
| monoclonal anti-rabbit STAT2 (#72604S) |  |
| polyclonal anti-goat AKT1/2 (N-19) | Santa Cruz, CA, USA |
| polyclonal rabbit anti-mouse/human phospho-STAT2 (Tyr 690) (sc-21689-R) |  |
| polyclonal rabbit anti-mouse/human STAT2 (sc-950) |  |
| monoclonal mouse anti-mouse/human GAPDH (sc-32233) |  |
| polyclonal goat anti-rabbit Immunoglobulins/HRP (P0448) | DAKO, Hamburg,  Germany |
| polyclonal goat anti-mouse Immunoglobulins/HRP (P0447) |  |

Table S5: gRNA Oligonucleotides

| gRNA | Sequence (5’🡪3’) |
| --- | --- |
| Oligo 1a STAT1 | CACCGGCAGGAAAACATTTACCTCT |
| Oligo 1b STAT1 | AAACAGAGGTAAATGTTTTCCTGCC |
| Oligo 2a STAT1 | CACCGGTGGCAGTAATCTCGATTCA |
| Oligo 2b STAT1 | AAACTGAATCGAGATTACTGCCACC |
| Oligo 3a STAT1 | CCGACGGTTAGAAGGTGGGGCCAAA |
| Oligo 3b STAT1 | AAACCGGGGTGGAAGATTGGCAGCC |
| Oligo 4a STAT1 | CCTGTGACTACCTCTCACGGACAAA |
| Oligo 4b STAT1 | AAACAGGCACTCTCCATCAGTGTCC |
| Oligo 1a STAT2 | CACCGGCTTCAGTAGCTGCCGAAGG |
| Oligo 1b STAT2 | AAACCCTTCGGCAGCTACTGAAGCC |
| Oligo 2a STAT2 | CACCGGAGTCACATGCTTCGGTATA |
| Oligo 2b STAT2 | AAACTATACCGAAGCATGTGACTCC |
| Oligo 3a STAT2 | CACCGGAGGGTCCGTATGGGGTGCC |
| Oligo 3b STAT2 | AAACGGCACCCCATACGGACCCTCC |
| Oligo 4a STAT2 | CACCGGTCCGCTCTTGGAGTGGGGG |
| Oligo 4b STAT2 | AAACCCCCCACTCCAAGAGCGGACC |

Table S6: ChIP antibodies

| Antibody | Company |
| --- | --- |
| Anti-Histone H3 (acetyl K9) | Millipore, Darmstadt, Germany |
| anti-Histone H3 (tri-methyl K4) | Abcam, Cambridge, UK |
| anti-Histone H3 (acetyl K27), |  |
| rabbit anti-mouse IgG |  |
| anti-Histone H3 (tri-methyl K27) | Diagenode, Liège, Belgium |

Table S7: ChIP-PCR Primer

| Target gene | Forward primer (5’🡪3’) | Reverse primer (5’🡪3’) |
| --- | --- | --- |
| *Stat1* | GGACGCCCTCTAGCCTTTTT | GTGTCCCAAGTGGGTCTGAG |
| *Stat2* | TACAGGCCCCGAAAGGGT | GCCTTGTCTACAGGGGCATC |
| *Irf1* | CTTCGCCGCTTAGCTCTACA | CTCCTGCGCGCCCATT |
| *Irf9* | GGTGCTACTGCTGACTGAGG | TACCTGAGCCGCTGAAAAGG |
|  |  |  |

Table S8: GSEA of a set of 45 Interferon Stimulated Genes (ISG)

**Table S9: Statistics for the enrichment analysis of transcription factor binding sites (TFBS) in acetylation (H3K9ac) peaks.**

The statistical data indicate that peaks with higher H3K9me3 values in Jak2V617F cells are enriched for Stat1 motifs, which are only a subset of peaks with higher H3K9ac in Jak2V617F than in EV. A similar pattern is observed for Irf1 and Irf8 motifs.
