# Supplementary figures and images for: Differential roles of STAT1 and STAT2 in the sensitivity of JAK2V617F- vs. BCR-ABL-positive cells to interferon alpha
Source: J Hematol Oncol. 2019 Apr 2;12:36. doi: 10.1186/s13045-019-0722-9 (PMC6444528; doi:10.1186/s13045-019-0722-9)

Figure S1

murine STAT1 (chr1:52,119,622-52,161,865)

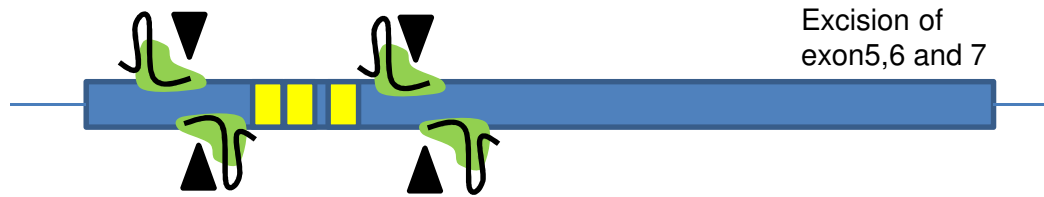

murine STAT2 (chr10:128,270,576-128,292,849)

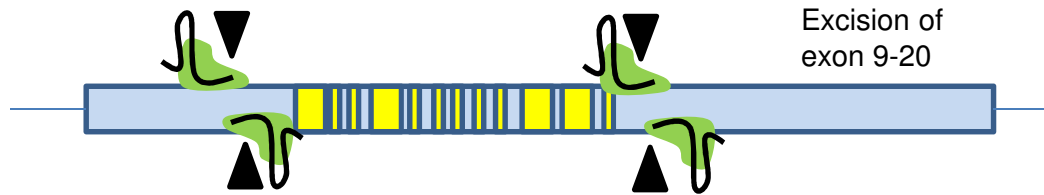

Supplement: Supplementary file 2 — Figure S1. Knockout of STAT1 or STAT2 with CRISPR/Cas9n technology. Four guide RNAs have been generated for STAT1 or STAT2 knockout in 32D-BCR-ABL and 32D-JAK2V617F cells, respectively. Excised exons are given. (PDF 19 kb) [file 13045_2019_722_MOESM2_ESM.pdf]

Figure S2

MTT assay: 72 hours

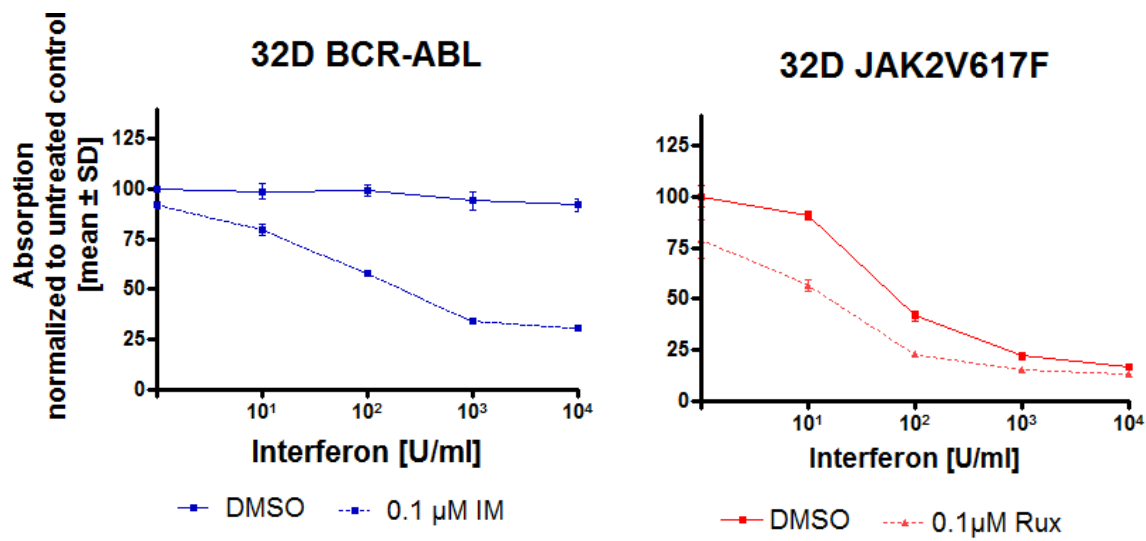

Supplement: Supplementary file 3 — Figure S2. MTT assay of 32D-BCR-ABL and 32D-JAK2V617F cells treated with IFNa. 32D-BCR-ABL-(blue) and 32D-JAK2V617F-(red) positive cells were treated with IFNa (0–104 U/ml) alone (continuous lines) or in combination with 0.1 μM imatinib (IM) or ruxolitinib (Rux) (dotted lines) for 72 h and the viability was measured by MTT. Viability was normalized to the untreated control and mean values ± SD are depicted. The respective 32D cells were WEHI starved for 24 h before starting the experiments. Experiments were performed in triplicate and conducted three times. (PDF 27 kb) [file 13045_2019_722_MOESM3_ESM.pdf]

Figure S3

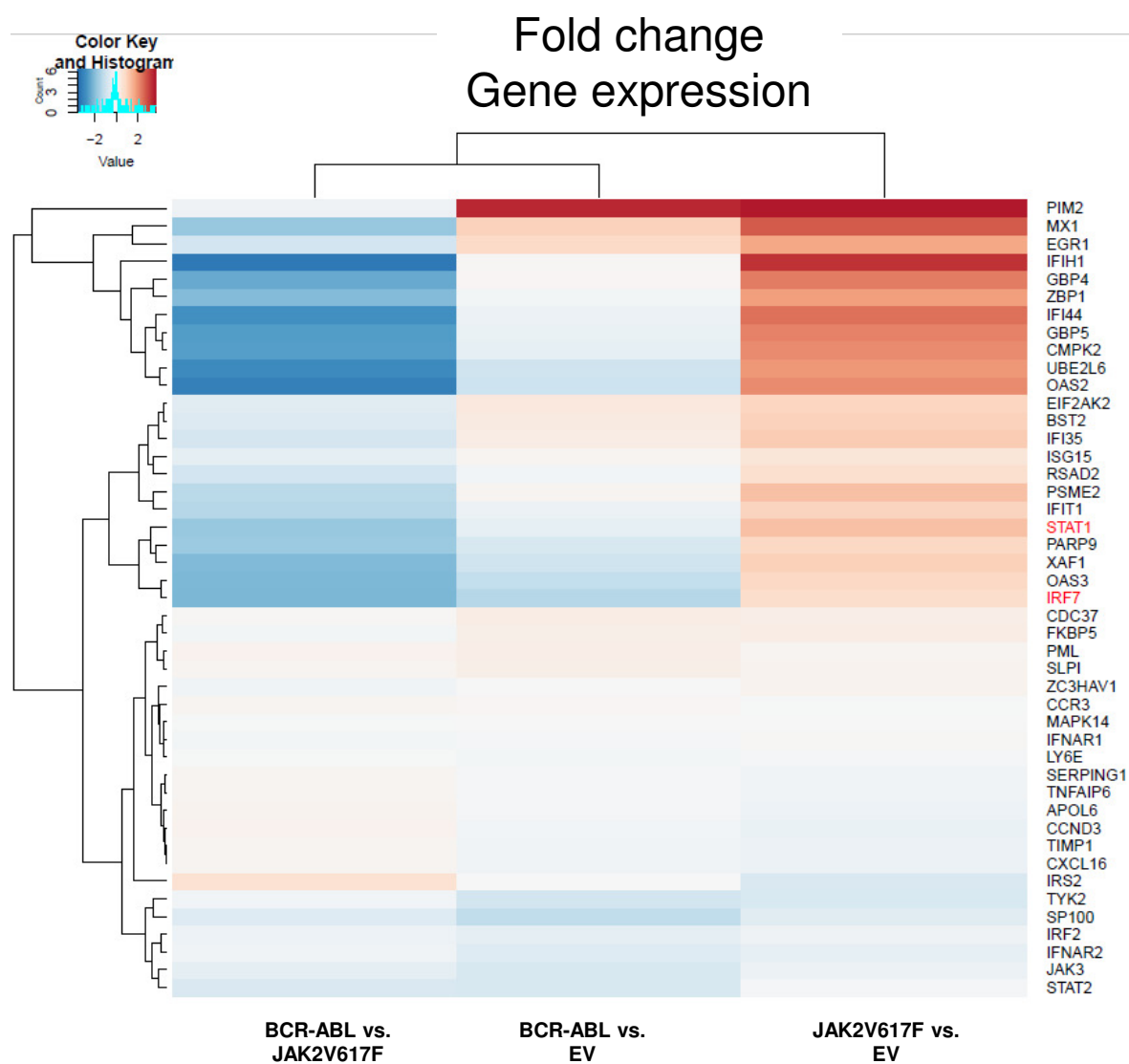

Supplement: Supplementary file 4 — Figure S3. BCR-ABL reduces ISG expression in 32D cells. Gene expression microarray analysis of 32D-EV, 32D-BCR-ABL, or 32D-JAK2V617F cells. Fold change of gene expression is shown, depicting downregulation of the analyzed gene in blue and upregulation in red. (PDF 134 kb) [file 13045_2019_722_MOESM4_ESM.pdf]

Figure S4

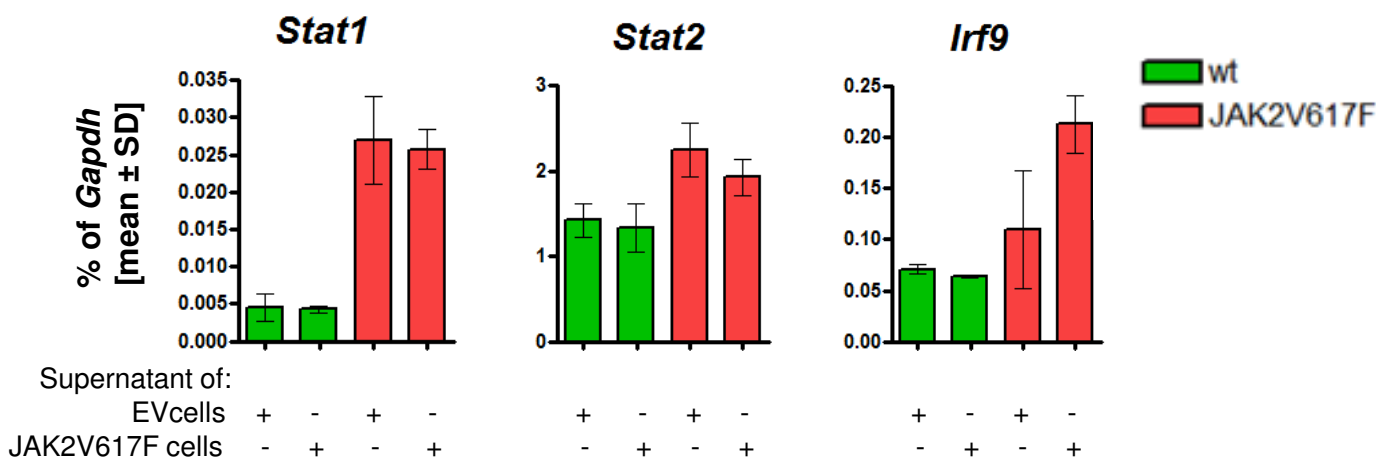

Supplement: Supplementary file 5 — Figure S4. Effect of extrinsic soluble factors on gene expression in 32D-EV- or 32D-JAK2V617F-positive cells. Supernatant of WEHI-starved 32D-EV- or 32D-JAK2V617F-positive cells was generated overnight, and after removal of the cells, fresh EV (green) or JAK2V617F-(red) positive cells were incubated with the supernatant for 2 h prior to RNA extraction to analyze the expression of IFN target genes. Mean ± SD values are shown as % of Gapdh. Independent experiments were performed three times and in triplicate, respectively. (PDF 25 kb) [file 13045_2019_722_MOESM5_ESM.pdf]

Figure S5

A

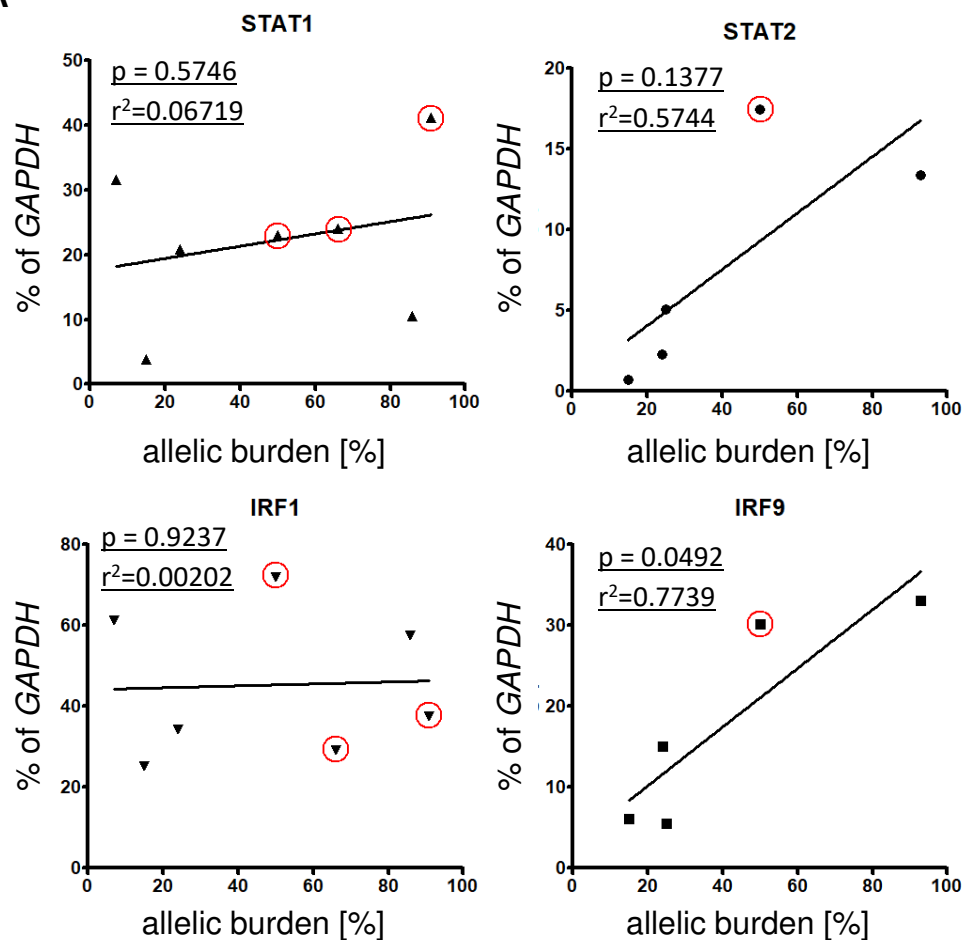

B

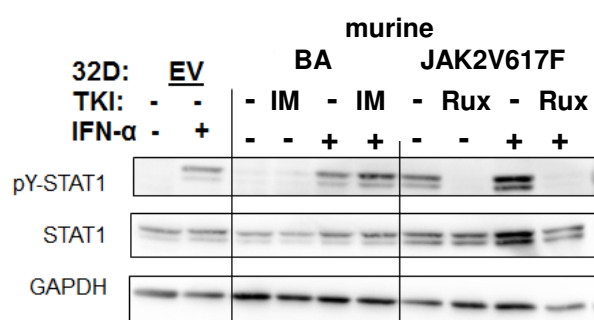

Supplement: Supplementary file 6 — Figure S5. Correlation of ISG expression and JAK2V617F allelic burden and Western blot of 32D EV, BCR-ABL, or JAK2V617F cells. A, ISG expression (% of GAPDH) and JAK2V617F allelic burden (in %) were plotted against each other and a significant correlation was only found for IRF9 (p = 0.0492). Samples with additional mutations (TET2, ASXL1, or EZH2) were highlighted with a red circle. B, Western blot analysis of pY-STAT1 and overall STAT1 protein after TKI and/or IFNa treatment (4 h) of 32D EV (only −/+ IFNa), BCR-ABL, or JAK2V617F cells. All cell lines were starved of WEHI (source of IL-3) for 24 h before treatment. GAPDH served as the loading control. The same Western blot is shown in Fig. 2c lacking 32D EV cells. (PDF 74 kb) [file 13045_2019_722_MOESM6_ESM.pdf]

Figure S6  
**32D STAT1 KO**

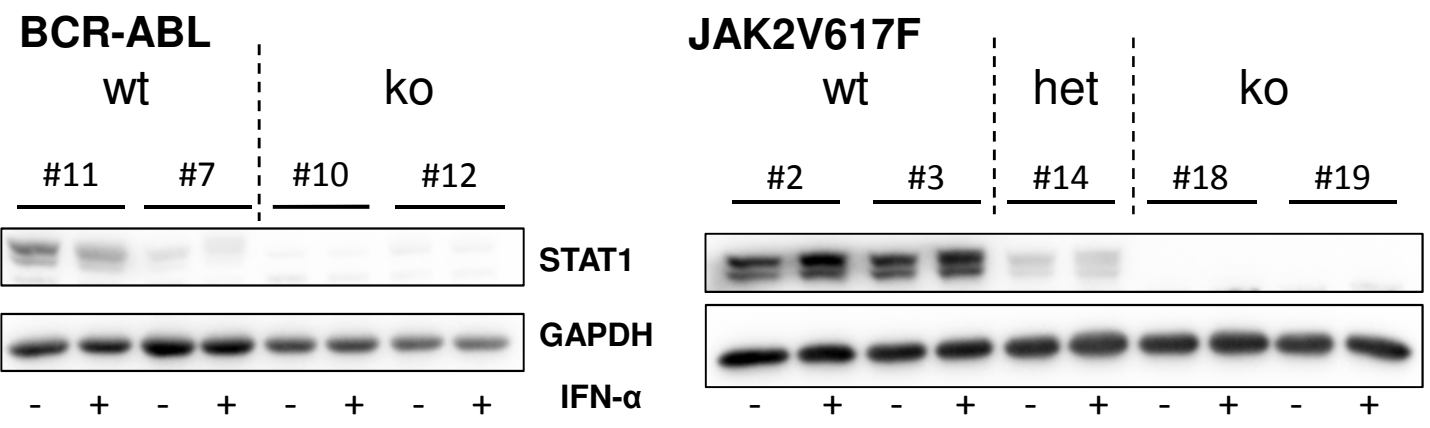

**32D STAT2 KO**

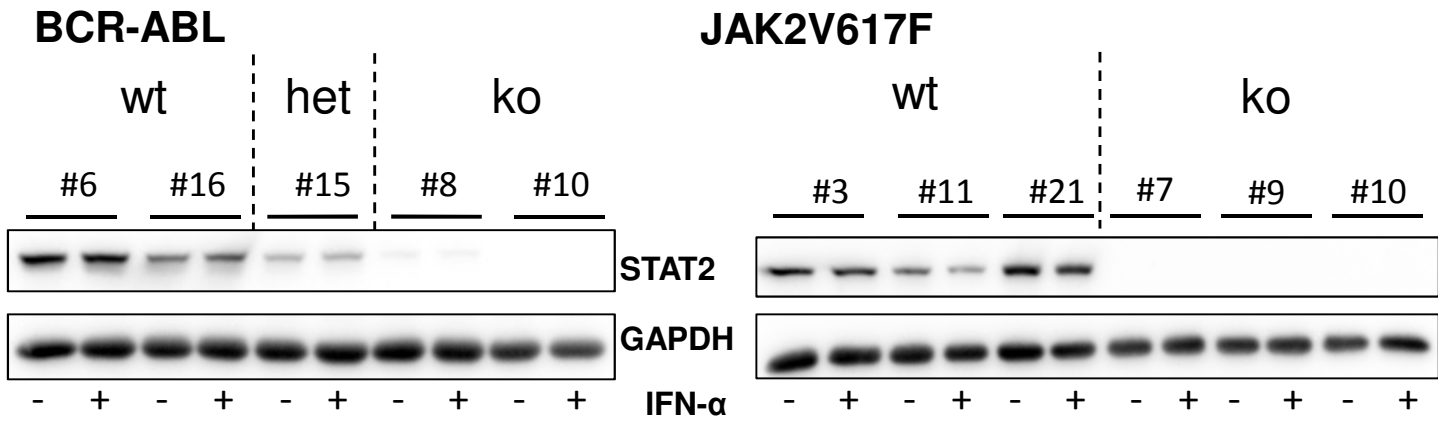

Supplement: Supplementary file 7 — Figure S6. Confirmation of successful STAT1 or STAT2 knockout. Western blotting of several 32D-BCR-ABL or 32D-JAK2V617F STAT1 or STAT2 knockout clones. STAT2 antibody was used to confirm the knockout, and GAPDH served as the loading control. 32D cells were WEHI starved for 24 h before starting the experiment. wt – wild-type clones, ko – knockout clones, het – presumed heterozygous clones (PDF 134 kb) [file 13045_2019_722_MOESM7_ESM.pdf]

Figure S9a

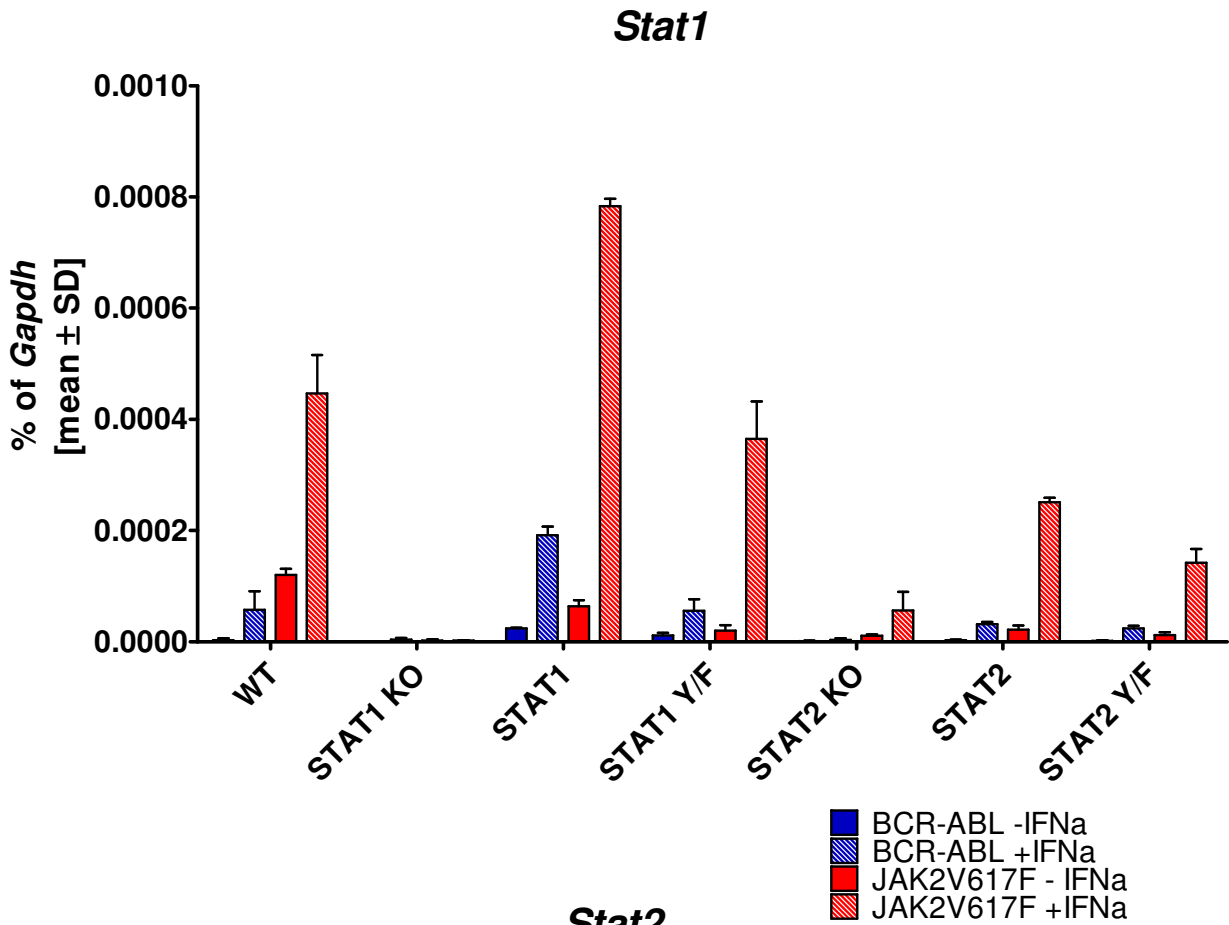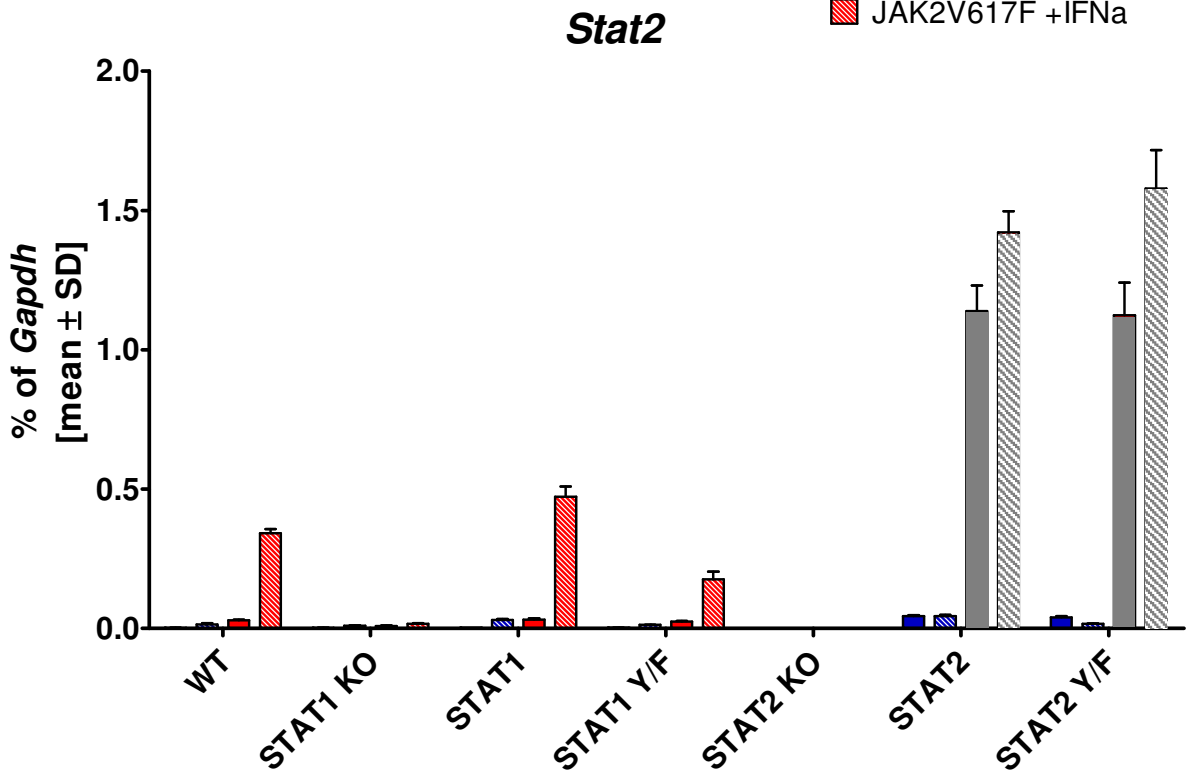

Supplementary Figure 9b

*Irf7*

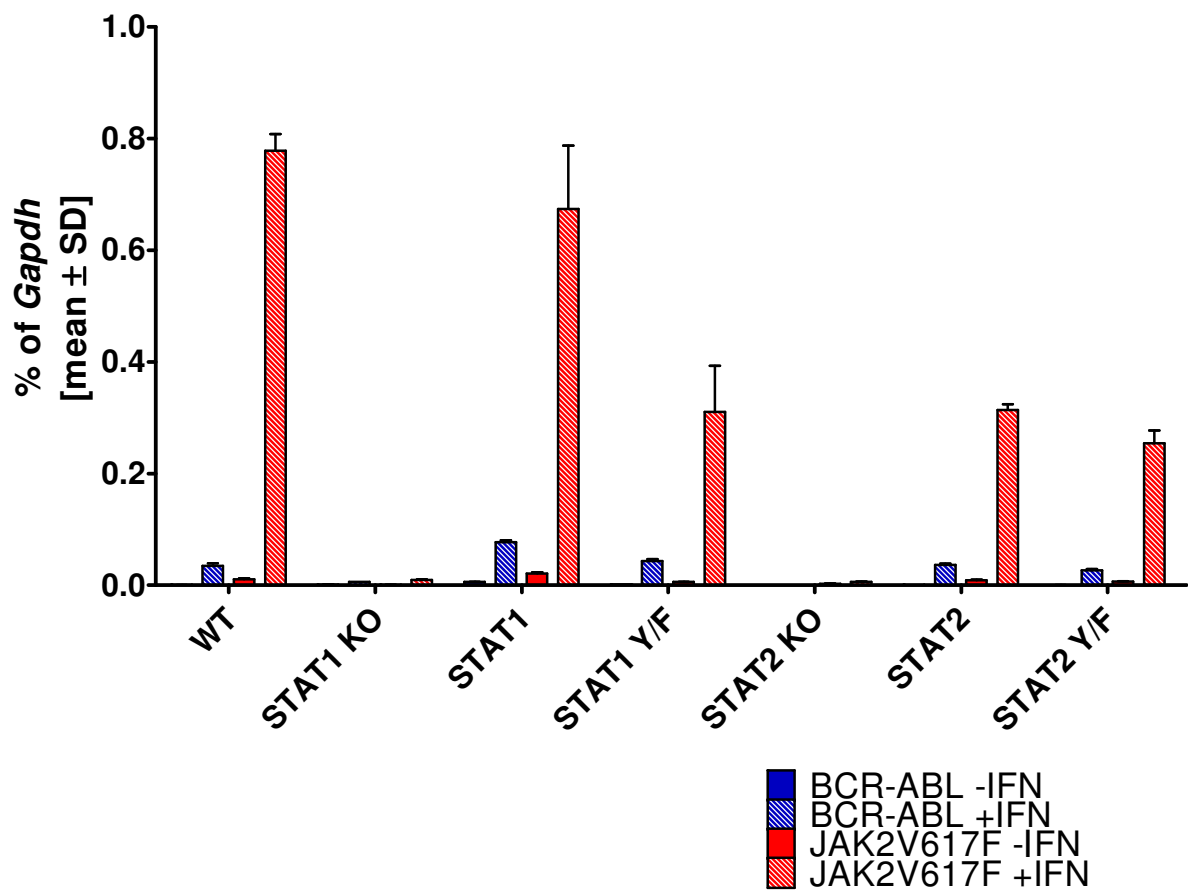

*Irf9*

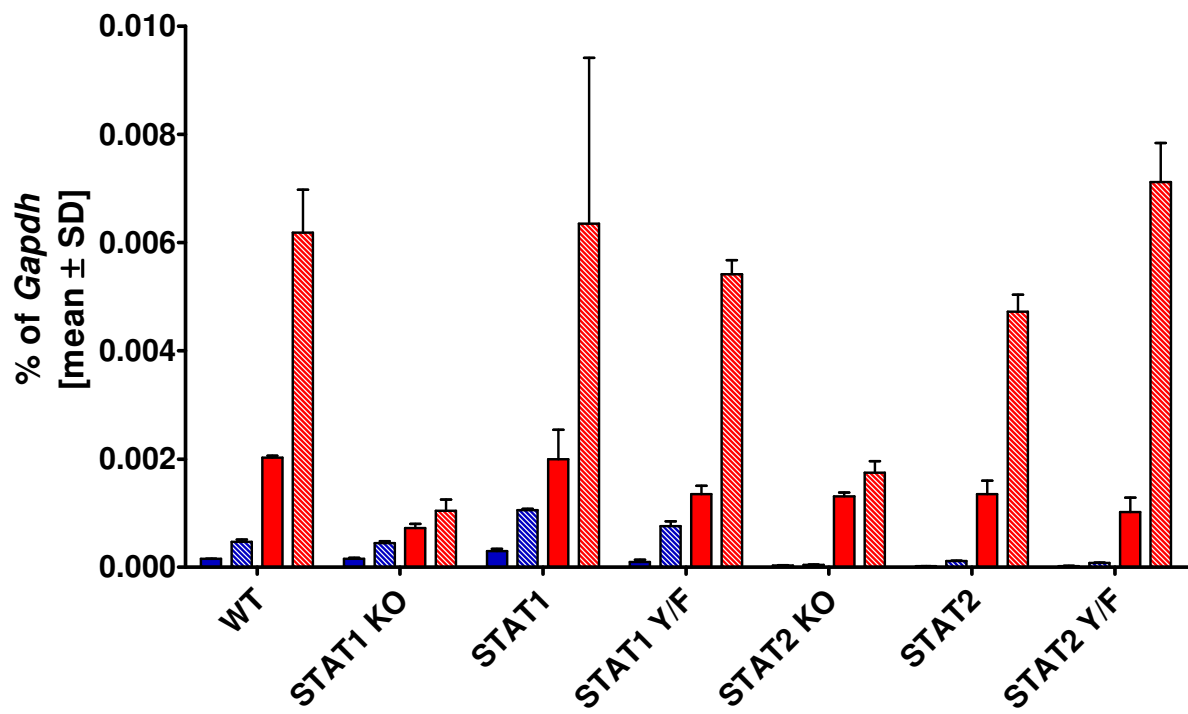

Supplement: Supplementary file 8 — Figure S9. Full RT-qPCR panels of tested ISGs. Illustration of the RT-qPCR results of 32D-BCR-ABL- and 32D-JAK2V617F-WT or -STATko or -STAT1(Y/F) and STAT2(Y/F) reconstituted cell clones treated with IFNa (100 U/ml) or left untreated (triplicate), corresponding to the data given in Figs. 3f and 4d. (a) Stat1 and Stat2, (b) Irf7 and Irf9. Stat2 qPCR primer detected the ectopically expressed Stat2 mRNA, explaining the strong upregulation, and endogenous Stat2 can thus not be evaluated in the reconstituted experiments (gray bars). Independent experiments were performed three times. (PDF 56 kb) [file 13045_2019_722_MOESM8_ESM.pdf]

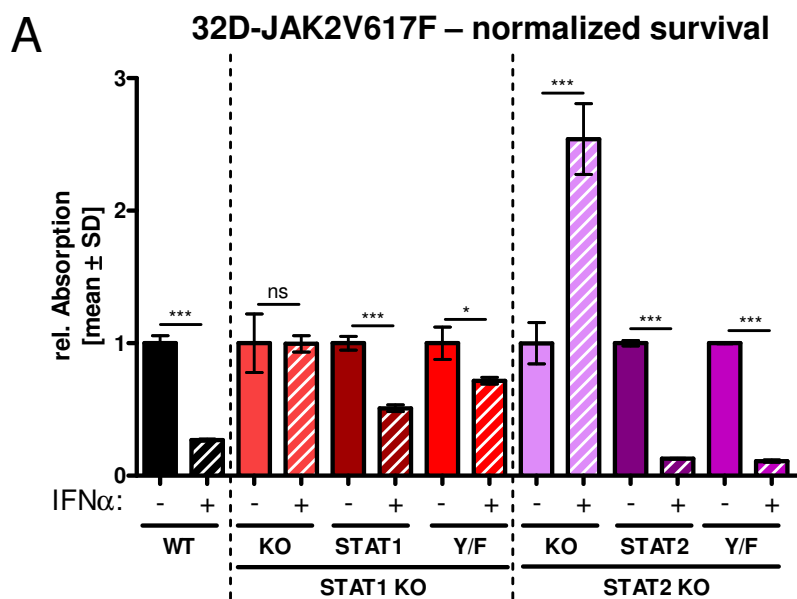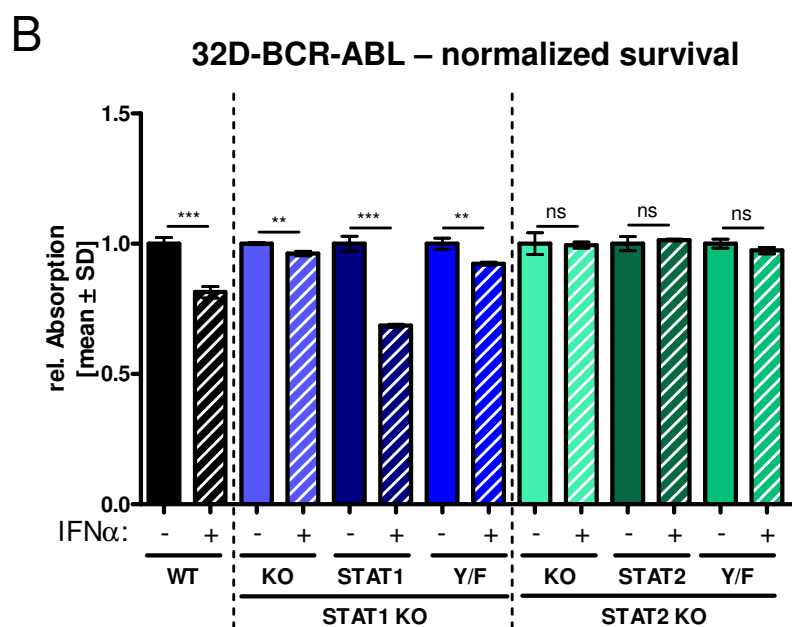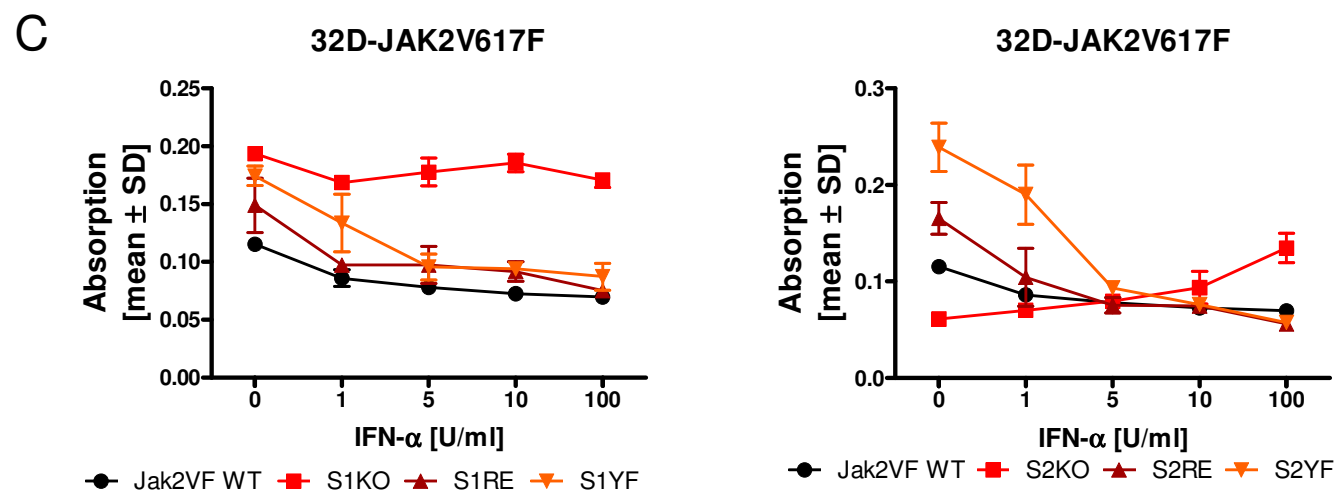

Supplement: Supplementary file 9 — Figure S7. Comparison of CRISPR/Cas9 manipulated 32D cell lines treated with 100 U IFNa in survival and titration of lower IFNa dosages. Indicated (A) 32D-BCR-ABL and (B) 32D-JAK2V617F cell lines were analyzed in an MTT assay and treated with 100 U IFNa for 72 h (abstracted from Fig. 4a, b). Absorption was normalized to untreated control cells and statistically analyzed using a t test. Mean values ± SD are indicated. *p < 0.05, **p < 0.01, ***p < 0.001. C, 32D-JAK2V617F cells, -S1ko and S2ko cells, reconstituted with STAT1 (S1RE), STAT1Y701F (S1YF), STAT2 (S2RE), or STAT2Y689F were treated with IFNa (0, 1, 5, 10 and 100 U/ml) for 72 h, and the viability was measured by MTT. Mean values ± SD are depicted. Independent experiments were performed three times and in triplicate, respectively. The respective 32D cells were WEHI starved for 24 h before starting the experiments. (PDF 26 kb) [file 13045_2019_722_MOESM9_ESM.pdf]

Figure S10

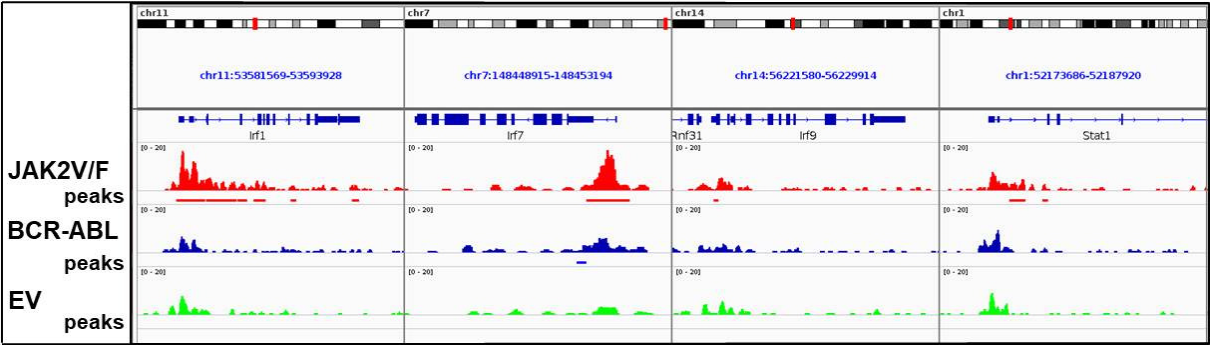

Supplement: Supplementary file 11 — Figure S10. H3K9 acetylation profile at ISGs. The ChIP-seq acetylation was visualized with the Integrative Genomics Viewer (IGV). Shown are the H3K9 acetylation peaks at the genomic regions of Irf1, Irf17, Irf9, and Stat1 in 32D-JAK2V7F (JAK2V617F) (red), 32D-BCR-ABL (blue), and 32D-EV (green). (PDF 108 kb) [file 13045_2019_722_MOESM11_ESM.pdf]
