# Supplementary material for: Differential roles of STAT1 and STAT2 in the sensitivity of JAK2V617F- vs. BCR-ABL-positive cells to interferon alpha
Source: J Hematol Oncol. 2019 Apr 2;12:36. doi: 10.1186/s13045-019-0722-9 (PMC6444528; doi:10.1186/s13045-019-0722-9)
Supplement: Supplementary file 10 — Figure S8. Illustration of pY-STAT1 in 32D-JAK2V617F cells in the absence of IFNa. STAT1 and pY-STAT1 stainings of Fig. 4c, shown with increased contrast. The arrows are indicating the pY-STAT1 bands in 32D-BCR-ABL- and 32D-JAK2V617F-STATko cells. (PDF 203 kb) [file 13045_2019_722_MOESM10_ESM.pdf]

Figure S8

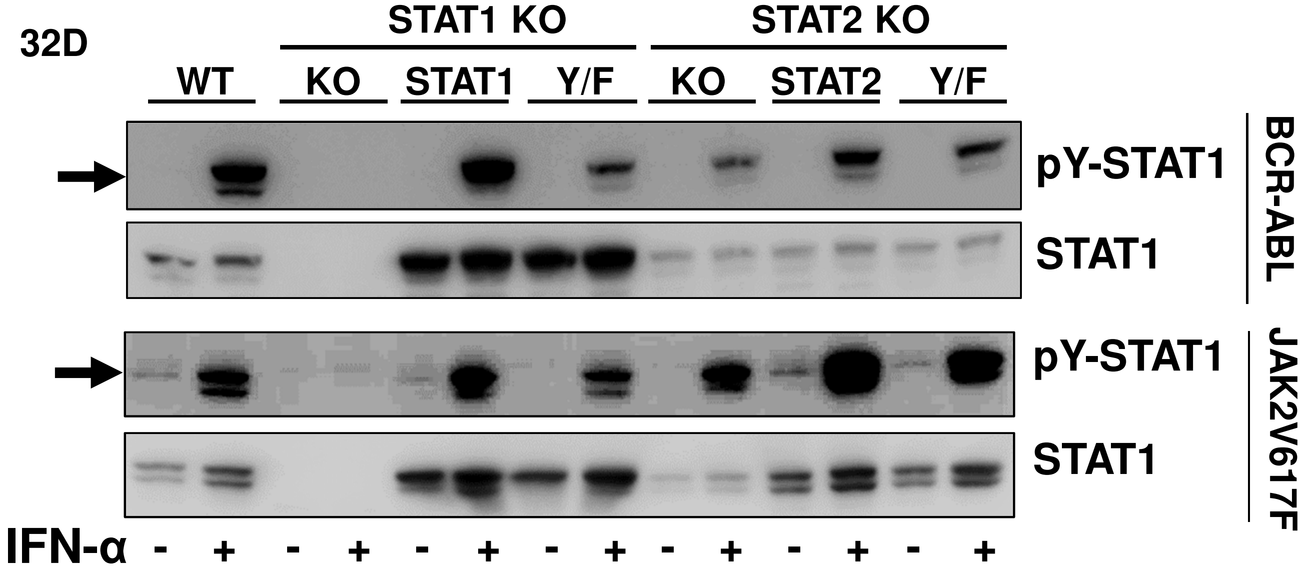
32D
STAT1 KO
STAT2 KO
WT
KO
STAT1
Y/F
KO
STAT2
Y/F
pY-STAT1
STAT1
BCR-ABL
pY-STAT1
STAT1
JAK2V617F
IFN-α
- + - + - + - + - + - + - +
